# Vasculogenic properties of adventitial Sca-1^+^CD45^+^ progenitor cells in mice: a potential source of vasa vasorum in atherosclerosis

**DOI:** 10.1038/s41598-019-43765-8

**Published:** 2019-05-13

**Authors:** Deborah Toledo-Flores, Anna Williamson, Nisha Schwarz, Sanuja Fernando, Catherine Dimasi, Tyra A. Witt, Thao M. Nguyen, Amrutesh S. Puranik, Colin D. Chue, Sinny Delacroix, Daniel B. Spoon, Claudine S. Bonder, Christina A. Bursill, Belinda A. Di Bartolo, Stephen J. Nicholls, Robert D. Simari, Peter J. Psaltis

**Affiliations:** 1 Vascular Research Centre, Lifelong Health Theme, South Australian Health and Medical Research Institute, Adelaide, Australia; 2 Adelaide Medical School, University of Adelaide, Adelaide, Australia; 3 Division of Cardiovascular Diseases, Mayo Clinic, Rochester, MN USA; 4 Centre for Cancer Biology, University of South Australia & SA Pathology, Adelaide, Australia; 5 University of Kansas School of Medicine, Kansas City, KS USA

**Keywords:** Transdifferentiation, Angiogenesis

## Abstract

The cellular origins of vasa vasorum are ill-defined and may involve circulating or local progenitor cells. We previously discovered that murine aortic adventitia contains Sca-1^+^CD45^+^ progenitors that produce macrophages. Here we investigated whether they are also vasculogenic. In aortas of C57BL/6 mice, Sca-1^+^CD45^+^ cells were localised to adventitia and lacked surface expression of endothelial markers (<1% for CD31, CD144, TIE-2). In contrast, they did show expression of CD31, CD144, TIE-2 and VEGFR2 in atherosclerotic *ApoE*^−/−^ aortas. Although Sca-1^+^CD45^+^ cells from C57BL/6 aorta did not express CD31, they formed CD31^+^ colonies in endothelial differentiation media and produced interconnecting vascular-like cords in Matrigel that contained both endothelial cells and a small population of macrophages, which were located at branch points. Transfer of aortic Sca-1^+^CD45^+^ cells generated endothelial cells and neovessels *de novo* in a hindlimb model of ischaemia and resulted in a 50% increase in perfusion compared to cell-free control. Similarly, their injection into the carotid adventitia of *ApoE*^−/−^ mice produced donor-derived adventitial and peri-adventitial microvessels after atherogenic diet, suggestive of newly formed vasa vasorum. These findings show that beyond its content of macrophage progenitors, adventitial Sca-1^+^CD45^+^ cells are also vasculogenic and may be a source of *vasa vasorum* during atherogenesis.

## Introduction

The formation of neovessels by angiogenesis and/or vasculogenesis is central to many physiologic and pathologic processes^1^. In angiogenesis, new vessels form as outgrowths from the endothelium of existing blood vessels, through the coordinated involvement of endothelial cell migration, proliferation and tube formation. In contrast, vasculogenesis occurs through the activity and differentiation of endothelial progenitor cells (EPCs) rather than established endothelium. Historically, much of this was thought to occur by the homing of circulating and/or bone marrow (BM)-derived EPCs to sites of tissue neovascularisation^2,3^; however, local tissue-resident progenitor cells that are not of BM origin have also been shown to contribute endothelial progeny to newly developing microvessels^4–6^. Angiogenesis and vasculogenesis are also supported by the production of soluble cytokines and growth factors by neighbouring cell populations that are not of endothelial lineage. These include myeloid angiogenic cells, such as M2-like (alternatively activated) macrophages^7–9^. Although different myeloid angiogenic cells undergo phenotypic changes in response to their surrounding microenvironment, they are generally not known to have endothelial differentiation capacity, and their involvement in neovascularisation is therefore regarded as paracrine.

The specialised microvascular network that supplies oxygen and nutrients to the walls of large blood vessels is known as the *vasa vasorum*. Arterial *vasa vasorum* comprise two subtypes, denoted as “interna” and “externa”, based on whether they arise from the intima/media or adventitia respectively^10^. Multiple lines of preclinical and clinical evidence have demonstrated intimate relationships between *vasa vasorum* density and atherosclerosis^10^. It follows that a better understanding of the cellular origins of *vasa vasorum* expansion is an important pursuit in the study of vascular physiology and atherogenesis. Among its cellular content, the adventitia contains different progenitor cell populations, which may be a local source of *vasa vasorum* formation^11^. One of the markers commonly used to identify progenitor cells in mouse adventitia, is stem cell antigen-1 (Sca-1)^11^. We recently identified that postnatal mouse arteries contain an adventitial Sca-1^+^CD45^+^ subpopulation that is enriched with adventitial macrophage progenitor cells (AMPCs)^12,13^. Given that resident macrophages are known to expand rapidly during neovessel formation in aortic ring studies^6,7^ and other angiogenic processes^14^, the current study investigated whether adventitial Sca-1^+^CD45^+^ progenitors may also have angiogenic or vasculogenic potential and contribute to *vasa vasorum* growth.

## Results

### Sca-1^+^CD45^+^ cells express endothelial markers in atherosclerotic but not healthy aorta

**Figure 1:**
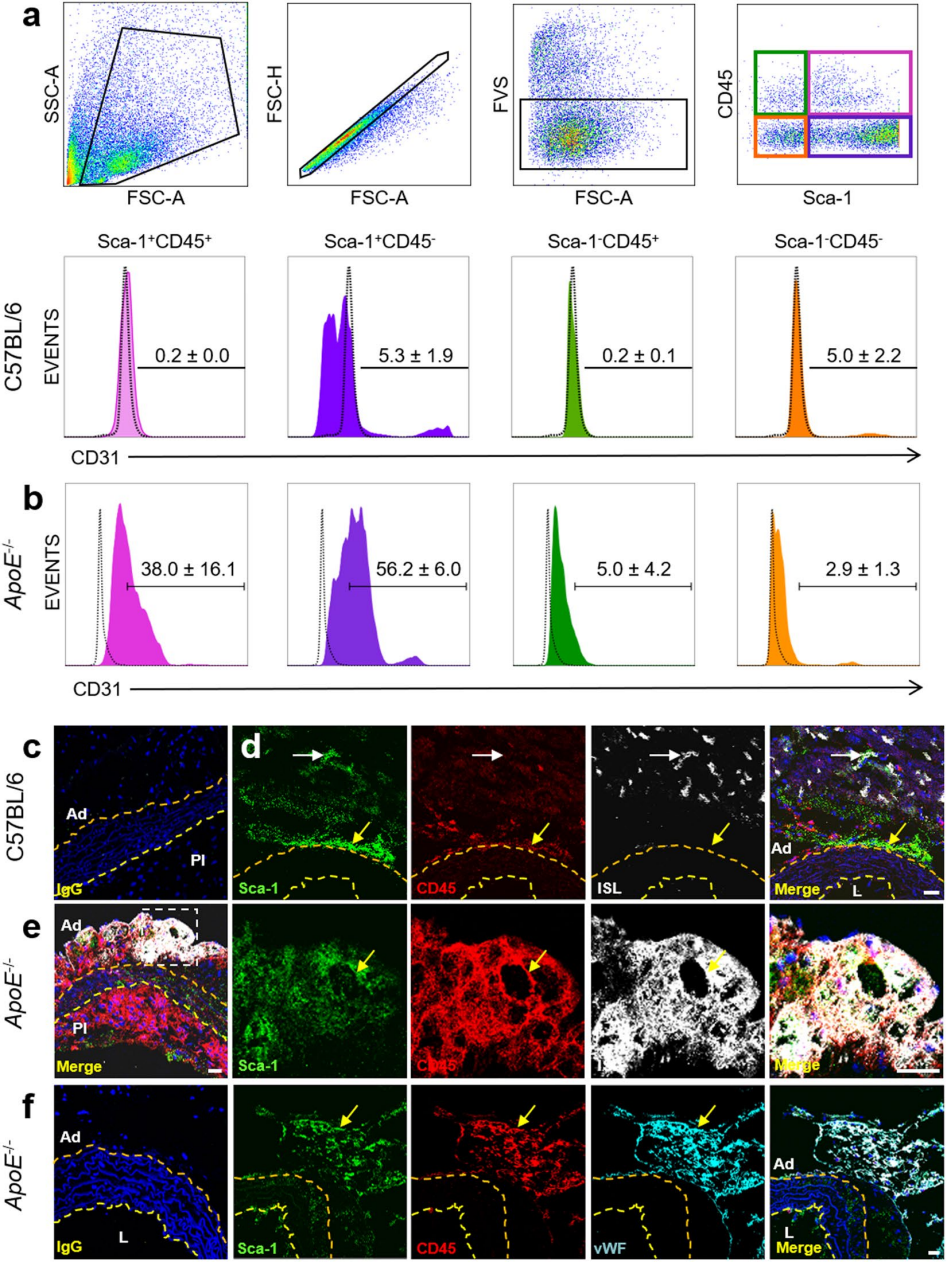
Expression of endothelial markers on Sca-1/CD45 subpopulations in C57BL/6 and *ApoE*^−/−^ aortas. (**a**) Flow cytometry gating strategy to identify four Sca-1/CD45 subpopulations of cells in aortic digests and their respective surface expression of CD31, from 12w C57BL/6 mice on chow diet. Percentage of CD31^+^ expression, relative to FMO negative control, for each subpopulation is summarised on histograms as mean ± sd from n = 3 mice. (**b**) Flow cytometry histograms showing mean ± sd % CD31 expression in the corresponding Sca-1/CD45 fractions from digests of 24w *ApoE*^−/−^ aortas after 16w of atherogenic diet to induce atherosclerosis. n = 3 mice. (**c–f**) Immunofluorescent staining and confocal microscopy of sections of aortic arch from 12w C57BL/6 mouse on chow diet (**d**) and from two different 24w *ApoE*^−/−^ mice on 16w atherogenic diet (**c,e–f**). (**c**) Merged image from IgG isotype control staining for Sca-1 (green), CD45 (red), ISL (white). (**d**) In C57BL/6 mice, Sca-1^+^CD45^+^ cells were present in adventitia but displayed minimal binding to ISL. ISL^+^ microvessels can be seen in peri-adventitial tissue only. White arrow indicates a peri-adventitial Sca-1^+^CD45^−^ cell with binding to ISL. Yellow arrow indicates an adventitial Sca-1^+^CD45^+^ cell closely apposed to the external elastic lamina, with no ISL binding (**e**,**f**). Dense co-localisation of Sca-1 and CD45 with ISL^+^ (white) (**e**) and vWF^+^ (cyan) (**f)** microvessels in adventitia of atherosclerotic *ApoE*^−/−^ aortas, with examples denoted by yellow arrows. Inset boxes in low magnification images correspond to adjacent high magnification images. IgG isotype control staining is also shown. Nuclei are counterstained blue with Hoechst. Broken yellow and orange lines indicate internal and external elastic lamina respectively. Ad, adventitia; L, lumen; Pl, plaque. Scale bar: 20 µm (white). Also see Supplementary Fig. 1.

We first used multicolour flow cytometry to compare expression of endothelial markers in four subpopulations of aortic cells gated based on Sca-1 and CD45 (Fig. 1a,b). CD31, CD144, TIE2, VEGFR2, CD106 (vascular cell adhesion molecule 1, VCAM-1) and LYVE1 were all expressed at low levels (<5% positive cells) overall in aortic digests from 12 week-old (12w) C57BL/6 mice, with highest expression seen in the Sca-1^+^CD45^−^ subpopulation which has previously been reported to contain endothelial and smooth muscle progenitor cells^15,16^. By comparison, the Sca-1^+^CD45^+^ population displayed very low co-expression of each of these markers, with <1% positive cells for each of CD31, CD144 and TIE2 (Fig. 1a, Table 1). As expected, the overall expression of each endothelial marker was increased in aortic digests from atherosclerotic *ApoE*^−/−^ mice compared to C57BL/6 mice, with the Sca-1^+^CD45^+^ fraction now containing >5% positive expression for each marker tested (Fig. 1b, Table 1).

**Table 1:** Endothelial marker expression on Sca-1/CD45 subpopulations in C57BL/6 and *ApoE*^−/−^ aortas.

Surface marker	Total	Sca-1^+^CD45^+^	Sca-1^+^CD45^−^	Sca-1^−^CD45^+^	Sca-1^−^CD45^−^
C57BL/6					
CD31^+^	1.9% (1.6–2.0)	0.3% (0.2–0.3)	5.2% (3.4–7.3)	0.2% (0.1–0.3)	4.8% (3.0–7.4)
CD144^+^	0.4% (0.4–0.5)	0.8% (0.6–1.2)	3.2% (2.5–6.2)	0.6% (0.3–1.0)	2.7% (1.3–6.7)
TIE2^+^	0.2% (0.1–0.3)	0.1% (0.1–4.4)	0.5% (0.4–0.5)	0.5% (0.1–2.9)	0.0% (0.0–0.0)
VEGFR2^+^	3.5% (3.4–5.2)	3.1% (1.4–16.9)	21.6% (20.9–23.4)	2.5% (0.9–10.5)	1.4% (1.1–1.9)
CD106^+^	3.9% (3.6–4.9)	2.8% (1.5–10.5)	19.4% (18.8–22.3)	2.1% (1.0–6.5)	2.0% (1.7–2.6)
LYVE1^+^	1.0% (0.7–1.2)	1.1% (0.5–6.7)	3.6% (2.9–3.8)	0.7% (0.2–2.9)	0.6% (0.5–0.7)
*ApoE* ^−/−^					
CD31^+^	26.6% (20.7–41.2)	47.2% (19.4–47.4)	52.9% (52.6–63.1)	5.3% (0.6–9.0)	2.4% (2.0–4.4)
CD144^+^	3.3% (3.0–10.5)	10.2% (3.2–10.5)	4.2% (3.3.–19.7)	3.3% (1.3–5.4)	1.1% (0.5–4.4%)
TIE2^+^	2.9% (1.9–3.5)	9.3% (8.2–15.4)	1.0% (0.6–2.0)	4.5% (1.4–5.3)	0.0% (0.0–0.1)
VEGFR2^+^	21.9% (14.9–31.4)	40.0% (26.3–41.5)	25.8% (23.8–52.7)	21.7% (10.2–24.7)	4.8% (1.2–6.2)
CD106^+^	13.2% (11.7–18.9)	21.0% (6.7–27.4)	27.0% (18.0–27.2)	6.7% (6.1–21.9)	7.3% (2.9–7.8)
LYVE1^+^	13.5% (12.9–16.5)	15.8% (10.3–32.2)	10.0% (9.8–25.8)	35.2% (1.1–41.8)	2.6% (1.9–6.8)

Shown are the median and range values for percent surface expression assessed by flow cytometry of six endothelial-related markers in aortic cell digests from n = 3 male 12w C57BL/6 mice fed chow-diet and n = 3 male 24w *ApoE*^−/−^ mice fed atherogenic diet for 16w, gated from the total viable population and the four Sca-1/CD45 subpopulations as shown in Fig. 1. Statistical comparisons were performed between corresponding cell populations from C57BL/6 and *ApoE*^−/−^ mice by Mann-Whitney test with all *P*-values being non-significant.

Using immunofluorescent staining and confocal microscopy, Sca-1^+^CD45^+^ cells were predominantly located in the adventitia of C57BL/6 aortas; these mice displayed a paucity of adventitial *vasa vasorum*, although microvessels were present in perivascular fat and connective tissue (Fig. 1d). In contrast, *ApoE*^−/−^ mice maintained on an atherogenic diet for 16w demonstrated transmural distribution of Sca-1^+^CD45^+^ cells across all three layers of their atherosclerotic aortas (Fig. 1e, Supplementary Fig. 1), and Sca-1 and CD45 were frequently co-expressed on *Griffonia simplicifolia* I-B4 isolectin^+^ (ISL^+^) and von Willebrand Factor^+^ (vWF^+^) *vasa vasorum*, and adventitial LYVE1^+^ lymphatics (Fig. 1e,f and Supplementary Fig. 1). These observations in *ApoE*^−/−^ aortas provided the first indication that Sca-1^+^CD45^+^ cells may have endothelial capacity and be involved in the formation of *vasa vasorum* when atherosclerosis is induced.

### Adventitial Sca-1^+^CD45^+^ cells possess endothelial plasticity and angiogenic capacity *in vitro*

**Figure 2:**
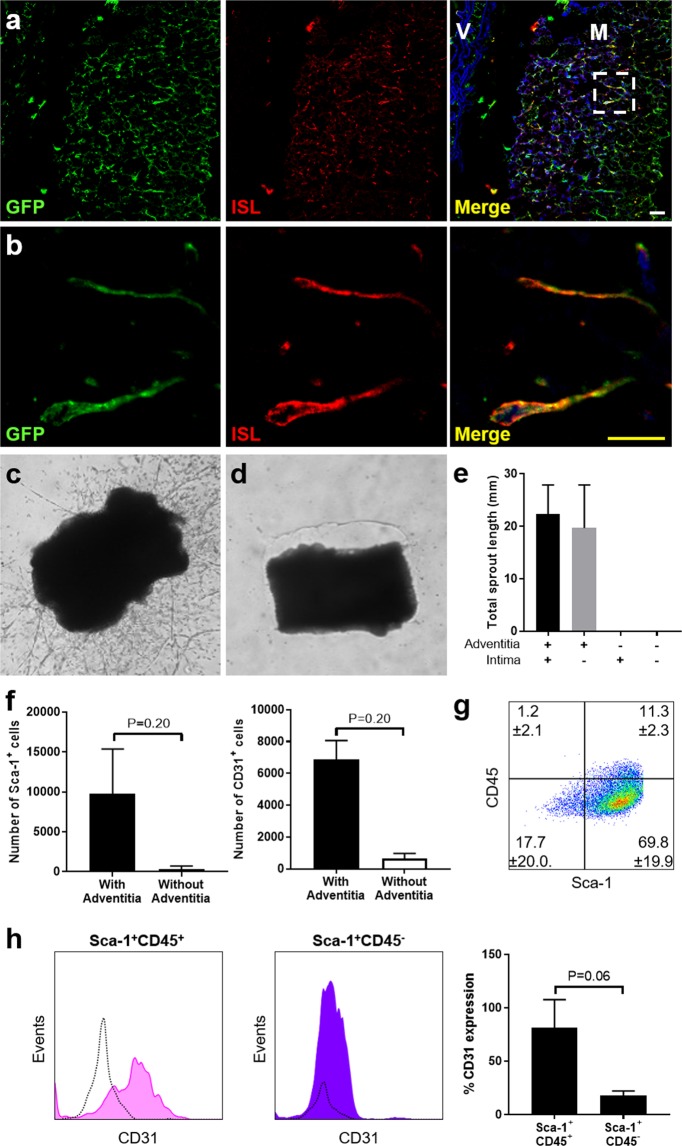
Contribution of adventitial Sca-1^+^ cells to *ex vivo* aortic ring sprouts. (**a**,**b**) Confocal microscopy images showing the binding of GFP^+^ (green) cells to ISL (red) following adventitial sprouting from aortic rings harvested from Ly6A (Sca-1)-GFP mice. Inset box in (**a**) corresponds to high magnification images in (**b**). Nuclei are counterstained blue with Hoechst. V, vessel wall; M, extra-vascular Matrigel. Scale bars: 10 µm (yellow), 20 µm (white). (**c**,**d**) Light microscopic images (x40) of sprouting from aortic rings with adventitia intact (**c**) and adventitia removed (**d**). (**e**) Graph showing the total length of adventitial sprouts grown from aortic rings from 12w C57BL/6 mice where the adventitia and/or intima were left intact (+) or removed/denuded (−). n = 3 donor mice per group. P-value was not significant by Friedman test. (**f**) Results from flow cytometry for the total number of outgrowing Sca-1^+^ and CD31^+^ cells in C57BL/6 aortic ring studies with and without adventitia. n = 3 donor mice per group. (**g**) Flow cytometry density plot for Sca-1 and CD45 expression from aortic ring adventitial outgrowths. (**h**) Representative histograms and graph depicting CD31 expression within the Sca-1^+^CD45^+^ and Sca-1^+^CD45^−^ populations growing out from C57BL/6 aortic rings. n = 5 donor mice. All quantitative data shown are mean ± sd. Statistical comparisons were performed using Mann Whitney tests in (**f**) and Wilcoxon matched-pairs signed rank test in (**h**).

We next studied aortas harvested from Ly6A-GFP (Sca-1-green fluorescence protein) transgenic mice^17^. GFP^+^ cells were predominantly located in the adventitia, and methlycellulose-based culture of aortic digests produced macrophage colony forming units (CFU-M) that were exclusively GFP^+^, consistent with our previous discovery that AMPCs are contained within the adventitial Sca-1^+^(CD45^+^) population^12,13^. *Ex vivo* aortic ring studies performed in Matrigel from these mice demonstrated that GFP^+^ cells of Sca-1^+^ origin participate in the process of angiogenic sprouting (Fig. 2a,b). We then confirmed that adventitial integrity is a prerequisite for this by showing that removal of the adventitia from C57BL/6 aortic rings eliminated sprouting, unlike intimal denudation which had little effect (Fig. 2c–e). To quantify the cellular composition of adventitial sprouts we scraped the Matrigel and performed collagenase digestion to separate the cellular outgrowths from the ring itself, and then analysed the resulting single cell suspensions by flow cytometry. In keeping with their failure to form angiogenic sprouts, aortic ring studies performed without adventitia had a lower content of both Sca-1^+^ and CD31^+^ cells than those with intact adventitia (Fig. 2f). Approximately 80% of the cellular make-up of aortic ring outgrowths was Sca-1^+^, with the majority of these cells lacking CD45 (69.8 ± 19.9% Sca-1^+^CD45^−^ and 11.3 ± 2.3% Sca-1^+^CD45^+^ of all viable cells, n = 6 donor mouse experiments with each using ≥ 3 aorta rings) (Fig. 2g). However, we observed a trend suggesting that CD31 was expressed on a higher percentage of outgrowing Sca-1^+^CD45^+^ cells than in the Sca-1^+^CD45^−^ subpopulation (Fig. 2h), and this was also the case for CD144, CD146, LYVE1, F4/80 and c-Kit (Supplementary Table 1). This aligned with our previous observation that although endothelial markers (e.g. CD31, CD144) were virtually absent from the adventitial Sca-1^+^CD45^+^ fraction in C57BL/6 aorta *in situ*, they became strongly co-expressed on these cells with *vasa vasorum* formation in atherosclerosis.

**Figure 3:**
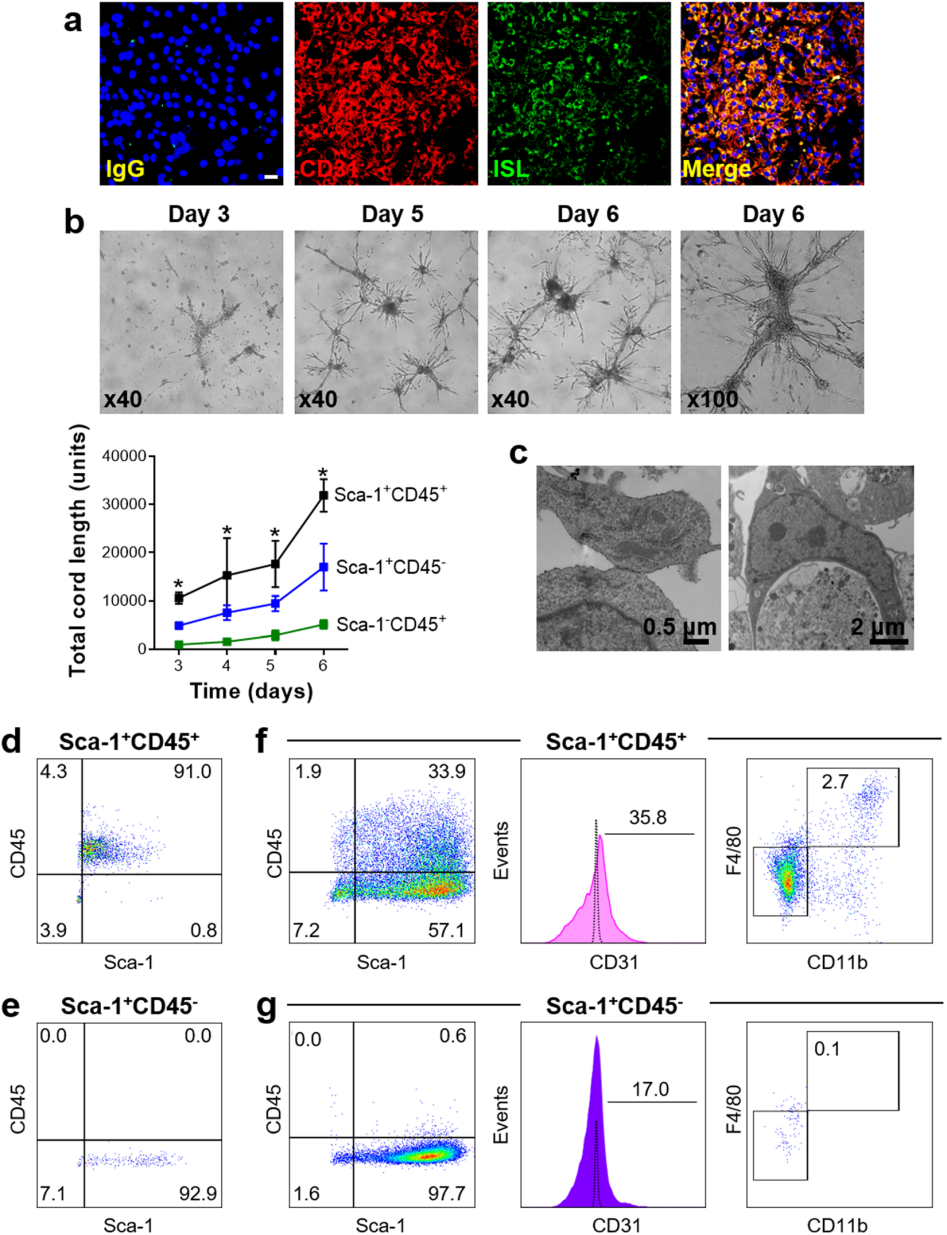
Endothelial plasticity and vascular cord forming capacity of adventitial Sca-1^+^CD45^+^ cells. (**a**) Immunofluorescent staining of adventitial Sca-1^+^CD45^+^ cells from C57BL/6 aorta after culture for 10 days in EGM-10 media containing VEGF. Note uniform expression of CD31 and binding to isolectin. Nuclei are counterstained blue with Hoechst. Also see Supplementary Fig. 3 for comparison to other inductive conditions. (**b**) Time course of vascular-like cord formation after plating Sca-1^+^CD45^+^ cells in Matrigel. Graph shows mean ± sd results from 3 independent experiments comparing cord formation from different Sca-1/CD45 subpopulations. Statistical comparisons were performed using Friedman tests at each time-point, with each P-value < 0.05. *P < 0.05 for Sca-1^+^CD45^+^ vs Sca-1^−^CD45^+^ by Dunn’s multiple comparisons test. (**c**) Transmission electron microscopy images from day 6 Sca-1^+^CD45^+^ well showing examples of intercellular adhesion (left) and phagocytosis (right). (**d**,**e**) Flow cytometry dot plots showing purity of freshly sorted Sca-1^+^CD45^+^ (d) and Sca-1^+^CD45^−^ (**e**) aortic fractions immediately before plating in Matrigel. (**f**,**g**) Representative dot plots and histogram showing expression of Sca-1, CD45, CD31, CD11b and F4/80 from cells obtained after cords had formed from starting Sca-1^+^CD45^+^ (**f**) and Sca-1^+^CD45^−^ (**g**) populations. Also see Table 2 and Supplementary Figs 2–6. Scale bar: 20 µm (white).

To interrogate more directly the vasculogenic potential of adventitial Sca-1^+^CD45^+^ cells, we sorted this population to high purity from digested C57BL/6 aortas (Supplementary Fig. 2). In contrast to the Sca-1^+^CD45^−^ fraction, freshly isolated Sca-1^+^CD45^+^ cells again displayed negligible CD31 expression (Supplementary Fig. 2). Moreover they did not acquire CD31 when cultured *in vitro* for 10d under basal conditions (RPMI/10% Fetal Calf Serum) or in the presence of interleukin-4 (IL-4) and granulocyte-macrophage colony-stimulating factor (GM-CSF), which we previously used to induce their differentiation into CD11c^+^ myeloid cells (Supplementary Fig. 3)^13^. Macrophage colony-stimulating factor (M-CSF), which directs these cells to an F4/80^+^CD115^+^ macrophage phenotype^13^, also resulted in only limited CD31^+^ expression (Supplementary Fig. 3). However, culture in endothelial growth medium (EGM) supplemented with vascular endothelial growth factor (VEGF) resulted in the appearance of endothelial-like colonies after 7–10 days, which formed a homogeneous, confluent cell layer with cobble-stone morphology between 14 and 21 days. These cells stained uniformly for CD31 and displayed binding to *Griffonia simplicifolia* I-B4 isolectin (Fig. 3a, Supplementary Fig. 3).

In Matrigel-based assays, freshly isolated Sca-1^+^CD45^+^ cells showed a time-dependent, intrinsic capacity to produce three-dimensional vascular structures, to a significantly greater extent than their Sca-1^−^CD45^+^ and Sca-1^−^CD45^−^ counterparts (Fig. 3b, Supplementary Fig. 4). These angiogenic cords took several days to form and displayed dense micro-sprouting at branch-points, not typical of the smooth cords produced within one day by human umbilical vein endothelial cells (HUVECs) (Supplementary Fig. 4). Matrigel-based co-culture delineated the ability of Sca-1^+^CD45^+^ cells to align with the early cords formed by HUVECs, before integrating into these structures and ultimately forming their own consolidated, linear connections after HUVEC cords had become fragmented (Supplementary Fig. 4). Transmission electron microscopy was used to more closely examine the vascular-like networks produced by aortic Sca-1^+^CD45^+^ cells in Matrigel. This revealed mixed presence of phagocytic cells and cells that were interconnected by adhesions (Fig. 3c), suggesting that they had produced heterogeneous progeny comprising macrophage and endothelial-like progeny.

We therefore performed further analysis of the cellular composition of the vascular-like networks. Day 7 cords were retrieved from Matrigel by scraping, digested with collagenase and then the resulting single cell suspensions were immunostained for flow cytometry (Fig. 3d–g, Table 2). This firstly revealed that the majority of Sca-1^+^CD45^+^ cells lost CD45 expression as they formed cords, suggesting that they largely transformed away from the myeloid lineage. Notably, the Sca-1^+^CD45^+^ fraction gave rise to a high percentage of Sca-1^+^CD45^−^ cells (Fig. 3f), but the converse did not occur to any extent (Fig. 3g). The Sca-1^+^CD45^+^ population was again found to generate CD31^+^ endothelial cells (Fig. 3f), along with a small percentage of cells expressing CD146, a marker associated with both endothelium and pericytes (Table 2). However, very few CD140b^+^ (PDGFRβ^+^) pericytes were produced by either Sca-1^+^ fraction under the culture conditions used (Table 2).

**Table 2:** Surface marker expression on cells isolated from vascular-like networks formed from Sca-1^+^CD45^+^ and Sca-1^+^CD45^−^ aortic cells in Matrigel.

	Sca-1^+^CD45^+^	Sca-1^+^CD45^−^	P-value
Sca-1^+^	95.6% (92.0–98.1)	86.0% (62.7–98.1)	0.250
CD45^+^	26.1% (16.3–29.1)	3.0% (0.6–4.7)	0.125
c-Kit^+^	14.4% (5.1–26.3)	5.3% (1.4–5.7)	0.250
CD31^+^	35.8% (11.3–43.9)	17.0% (6.8–17.2)	0.500
CD146^+^	5.3% (0.7–28.0)	2.7% (0.7–8.8)	0.625
CD140b^+^	1.8% (0.2–2.8)	0.0% (0.0–0.1)	0.125

Shown are the median and range values for percent expression of different surface markers expressed by cells that were obtained from the Matrigel angiogenic cord assay, seven days after culturing freshly sorted Sca-1^+^CD45^+^ or Sca-1^+^CD45^−^ aortic cells from 12w C57BL/6 mice (n = 3–4 different experiments, with each using cells sorted from n = 8–10 pooled aortas). Statistical comparisons were performed by Wilcoxon matched-pairs signed ranks test.

As expected, Sca-1^+^CD45^+^ cells did produce a small population of macrophages in Matrigel (Fig. 3f), which were not observed from Sca-1^+^CD45^−^ cells (Fig. 3g): median % of Lin^−^CD45^+^CD11b^+^F4/80^+^ macrophages was 3.4% (range 2.4–3.5%) from Sca-1^+^CD45^+^ and 0.1% (0.0–0.1%) from Sca-1^+^CD45^−^ fractions (n = 3 experiments). To determine the distribution of macrophages relative to cords, we performed additional experiments using sorted adventitial cells from *Cx3cr1*^GFP/+^ mice, in which GFP signal enabled detection of CX_3_CR1^+^ progeny, that included macrophages. As shown in Supplementary Fig. 5, GFP^+^ cells were present in the vascular-like networks formed from the Sca-1^+^CD45^+^ but not the Sca-1^+^CD45^−^ fraction, specifically at the branch-point intersections of cords but not in or along the cords themselves. To establish the importance of macrophage progeny to their cord-forming capacity, Sca-1^+^CD45^+^ cells were also treated with liposomal clodronate which was added to the Matrigel every second day to deplete macrophage numbers. Although this had no effect on the total length of cords formed, it did appear to result in reduction of branching (Supplementary Fig. 6), consistent with the known ability of macrophages to support endothelial tip cell anastomosis^18^.

Collectively our observations from both *ex vivo* aortic ring sprouting and *in vitro* vascular cord formation indicate that adventitial Sca-1^+^CD45^+^ progenitors possess intrinsic endothelial plasticity and vasculogenic potential.

### Transcriptomic profiling of angiogenic and vasculogenic genes

Previously we reported the results of unbiased mRNA microarray analysis that compared Sca-1^+^CD45^+^, Sca-1^+^CD45^−^ and Sca-1^−^CD45^+^ cells from C57BL/6 aortas^13^. Re-interrogation of this data-set revealed that compared to Sca-1^−^CD45^+^ cells, Sca-1^+^CD45^+^ cells expressed higher transcript levels of at least fifty genes that are known to regulate angiogenesis and/or vasculogenesis (Table 3, Supplementary Fig. 7). Notable among these were: *Cd248* (endosialin), which is involved in developmental and tumour-associated neovascularisation^19^, and has been shown to be upregulated in atherosclerotic vessels of *ApoE*^−/−^ mice^20^; *Mmp2*, *Mmp3*, *Mmp14*, *Mmp23*, *Egfr*, *Pdgfra*, *Il33*, *Sox9* and *Cd34*, which were recently reported to be more highly expressed in tissue-resident EPCs than in mature endothelial cells^5^; *Ace* and *Agt*, which are members of the angiotensin-renin system; and various well-known growth factors, cytokines and chemokines that are pro-angiogenic (*Cxcl12*, *Csf1*, *Vegfa*, *Il6*) or lymphangiogenic (*Vegfc*). The same genes were not differentially expressed between Sca-1^+^CD45^+^ and Sca-1^+^CD45^−^ progenitor cells, indicating that both of these Sca-1^+^ subpopulations are transcriptionally primed to promote neovascularisation. Importantly, gene expression of mature endothelial markers (e.g. *Cd31*, *Vwf*, *Tie1* or *2*, *Enos*) was not significantly higher in Sca-1^+^CD45^+^ than Sca-1^−^CD45^+^ cells from C57BL/6 aortas, in keeping with our results from flow cytometry (Fig. 1a, Table 1).

**Table 3:** Angiogenic and vasculogenic gene expression in adventitial Sca-1^+^CD45^+^ progenitor cells.

Gene	Fold difference	FDR	Gene	Fold difference	FDR
*Cd248*	11.9	1.3E-05	*Rnase4*	2.5	8.3E-04
*Angptl1*	10.9	2.5E-05	*Angpt4*	2.4	2.2E-04
*Ccl11*	8.2	2.4E-05	*Serpinf1*	2.4	6.0E-04
*Cxcl12*	5.6	4.2E-04	*Tgfb3*	2.4	8.1E-04
*Timp1*	5.6	2.9E-05	*Timp3*	2.4	1.6E-03
*Pdgfra*	4.7	6.8E-05	*Ace*	2.4	1.2E-03
*Mmp3*	4.3	2.3E-04	*Thbs2*	2.3	2.5E-04
*Fst*	4.1	2.3E-04	*Angptl2*	2.2	7.7E-04
*Cd34*	4.0	5.8E-05	*Tek*	2.2	1.9E-02
*Il33*	3.8	7.7E-05	*Efna1*	2.1	2.0E-02
*Mmp2*	3.7	2.9E-04	*Igf1*	2.1	3.2E-04
*Ntrk2*	3.6	1.1E-04	*Adamts1*	2.0	3.6E-02
*Mmp23*	3.6	2.0E-04	*Angptl4*	1.9	2.9E-02
*Sox9*	3.5	2.4E-05	*Smo*	1.9	3.2E-03
*Csf1*	3.4	2.8E-05	*Ephb4*	1.8	3.3E-02
*Egfr*	3.4	1.4E-04	*Timp2*	1.8	3.8E-03
*Vegfc*	3.3	2.1E-04	*Col4a3*	1.7	1.5E-02
*Agt*	2.9	2.4E-04	*Tgfb2*	1.7	3.8E-03
*Angptl7*	2.8	8.8E-04	*Ang*	1.6	1.1E-02
*Anpep*	2.8	5.1E-04	*Erbb2*	1.6	1.5E-02
*Mmp14*	2.8	4.0E-03	*Il6*	1.6	3.1E-02
*Fn1*	2.6	8.1E-03	*Jag1*	1.6	3.1E-03
*Hey1*	2.6	8.6E-03	*Ptgs1*	1.5	5.0E-02
*Mdk*	2.6	3.1E-04	*Ptk2*	1.5	6.0E-03
*Cxcl1*	2.5	7.7E-04	*Vegfa*	1.5	2.0E-02

Shown are fifty genes that are known to be involved in angiogenesis and/or vasculogenesis and were found by microarray analysis to be more highly expressed by a factor of 1.5 or more in aortic Sca-1^+^CD45^+^ cells compared to Sca-1^−^CD45^+^ cells. FDR = false discovery rate. N = 3 donor experiments, with each experiment pooled from n = 6 12w C57BL/6 mice.

### Adventitial Sca-1^+^CD45^+^ cells adopt endothelial fate and form new vessels *in vivo*

**Figure 4:**
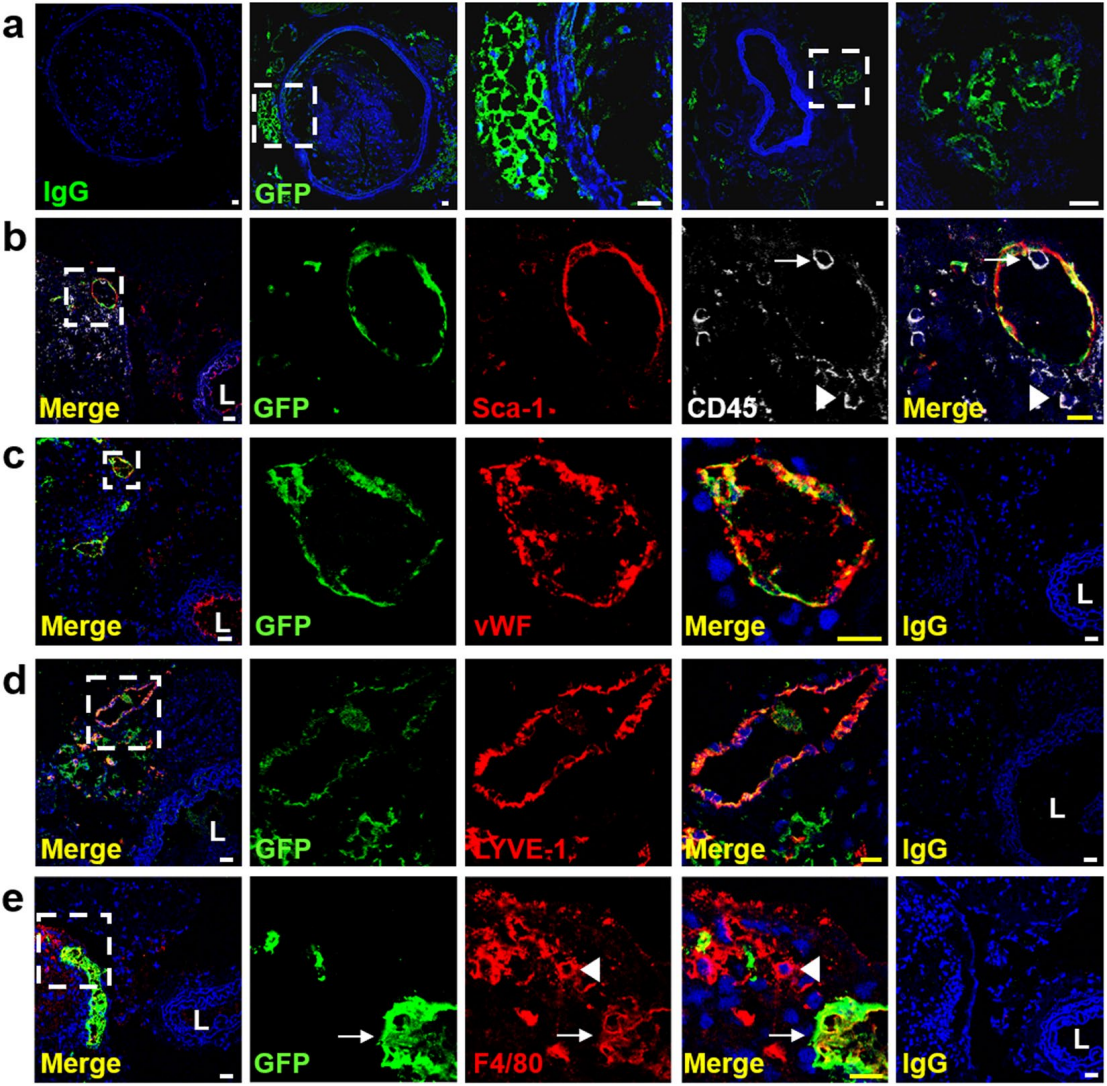
Formation of adventitial and peri-adventitial microvessels by Sca-1^+^CD45^+^ cells in atherosclerosis. (**a**) Confocal images of *ApoE*^−/−^ carotid arteries 16w after adventitial injection of aortic GFP^+^Sca-1^+^CD45^+^ cells and atherogenic diet. Note the presence of GFP^+^ cells forming lumen-containing microvascular structures and networks in adventitia and peri-adventitia. (**b–e**) Co-staining for GFP with Sca-1 and CD45 (**b**), vWF (**c**), LYVE1 (**d**) and F4/80 (**e**), showing that donor cells produced durable endothelial-lined microvessels in adventitia and peri-adventitia of atherosclerotic carotid artery, and also formed macrophages. Inset boxes correspond to adjacent high magnification images. In (**b**), the white arrow points to a GFP^−^ (host-derived) CD45^+^ leukocyte inside the lumen and adherent to the luminal surface of a well-formed GFP^+^ vascular structure suggesting integration with the host circulation. The white arrowhead in the same image indicates a cluster of host leukocytes around the outside of this neovessel, suggesting possible transmigration across it. IgG control staining is also shown for each set of images. Nuclei are counterstained blue with Hoechst. L, lumen. Scale bars: 10 μm (yellow), 20 μm (white).

We next tracked the fate of Sca-1^+^CD45^+^ cells during atherogenesis by isolating them from aortas of GFP donor mice and then injecting them into the carotid artery adventitia of 8w *ApoE*^−/−^ recipients^13^. Following 16w of atherogenic diet, GFP^+^ cells were found in and around the injected artery of all recipient mice (n = 6), but not in peripheral blood, remote tissues or contralateral carotid artery. In addition to giving rise to GFP^+^ macrophages (see previous publication^13^), we also detected networks of interconnected GFP^+^ cells and lumen-containing GFP^+^ structures in the carotid adventitia and peri-adventitia (Fig. 4a). These displayed preserved expression of Sca-1 (Fig. 4b) and stained for vWF (Fig. 4c) and LYVE1 (Fig. 4d), indicating that donor Sca-1^+^CD45^+^ cells had produced new microvessels and lymphatics. As seen in Fig. 4b, the presence of a GFP^−^ (host) CD45^+^ leukocyte adherent to the luminal surface of a GFP^+^ vessel was consistent with connection to the host circulation, while the clustering of host CD45^+^ cells just outside the same structure suggested that it may have supported leukocyte transmigration. Interestingly, we also found examples of complex adventitial and peri-adventitial GFP^+^ networks that expressed F4/80 (indicating macrophage content), but these were not found to stain for vWF (Fig. 4e).

**Figure 5:**
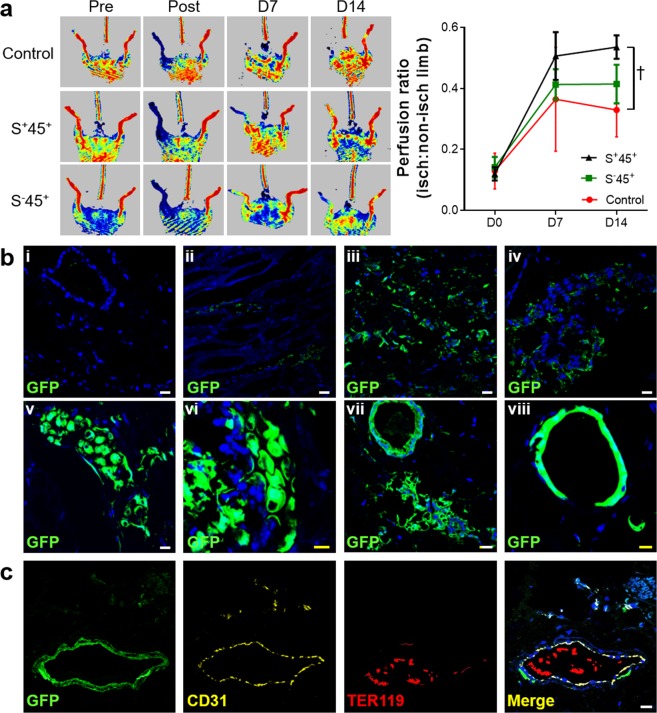
Vasculogenic properties of adventitial Sca-1^+^CD45^+^ cells in hindlimb ischaemia model. (**a**) Representative doppler perfusion images of C57BL/6 mice before and after hindlimb ischaemia surgery with intramuscular injection of cell-free Matrigel, aortic adventitial GFP^+^Sca-1^+^CD45^+^ (S^+^45^+^) or GFP^+^Sca-1^−^CD45^+^ (S^−^45^+^) cells. Graph summarises mean ± sd perfusion ratios of ischaemic:nonischaemic limb over time (n = 5–6 per group). *P* = 0.001 by Kruskal-Wallis test, with ^†^*P* < 0.01 for S^+^45^+^ vs control by Dunn’s multiple comparisons test. (**b**) GFP detection in gastrocnemius sections from ischaemic limb 14 days after injection of (i) Matrigel, (ii) Sca-1^+^CD45^−^, (iii) Sca-1^−^CD45^+^, (iv) Sca-1^−^CD45^−^ or (v-viii) Sca-1^+^CD45^+^ cells (four different recipient mice shown). (**c**) Example of a GFP^+^CD31^+^ blood vessel containing TER119^+^ erythrocytes in its lumen, 14 days after ischaemic surgery and injection of GFP^+^Sca-1^+^CD45^+^ cells. A representative merged image from IgG isotype control staining is shown in Supplementary Fig. 8. Nuclei are counterstained blue with Hoechst. Scale bars: 10 μm (yellow), 20 μm (white).

Finally, we used a model of hindlimb ischaemia to study the vasculogenic potential of adventitial Sca-1^+^CD45^+^ cells outside of their native environment of the artery wall. Recipient C57BL/6 mice were subjected to permanent ligation surgery of their left iliac artery and distal vessels before receiving intramuscular injections of cell-free Matrigel, GFP^+^ Sca-1^+^CD45^+^ cells, or other GFP^+^ Sca-1/CD45 subpopulations. Two weeks later, doppler imaging revealed that perfusion in the ischaemic limb was 50% higher in recipients of Sca-1^+^CD45^+^ progenitor cells than Matrigel control and this was accompanied by trends toward better clinical health and limb movement, (n = 5–6 per group) (Fig. 5a, Supplementary Fig. 8). Notably, injection of Sca-1^−^CD45^+^ leukocytes resulted in no significant benefit to hindlimb perfusion.

Qualitative analysis of immunostaining from sections of gastrocnemius muscle was then used to interrogate the fate of donor cells. Sca-1^−^CD45^+^ cells were only observed to be retained as clusters of individual cells (Fig. 5b). In contrast, each mouse that received Sca-1^+^CD45^+^ cells displayed robust and complex GFP^+^ networks and lumen-containing tubular structures (Fig. 5b). Among these structures, we found examples of CD31 expression, isolectin binding, connection to host vasculature and active cell proliferation (Ki67^+^) (Fig. 5c, Supplementary Fig. 8). Some tissue sections also contained isolated clusters of individual GFP^+^ cells that expressed the macrophage marker, MOMA-2, although these were rare and not in close proximity to GFP^+^ neovessels (Supplementary Fig. 8). Despite resulting in significant improvement in perfusion and the formation of new donor-derived blood vessels in the recipient hindlimbs, we were unable to demonstrate an overall increase in either CD31^+^ capillary or CD31^+^SMA^+^ arteriolar density in the tissue sections of Sca-1^+^CD45^+^ recipients compared to the Matrigel control group (Supplementary Fig. 9).

Together the results of these two adoptive transfer studies confirm that in addition to its content of macrophage progenitors^13^, the adventitial Sca-1^+^CD45^+^ subpopulation also produces mature endothelial cell progeny, that contribute to the formation of functional and durable neovessels *in vivo*.

## Discussion

The importance of the adventitia, and more specifically its *vasa vasorum* network, to vessel wall health and disease is well established^10,11,21–24^. Although knowledge of the developmental basis of *vasa vasorum* remains incomplete, existing data point to integral roles for different populations of mature and ancestral cells located within the adventitia. On the one hand, the angiogenic proliferation and sprouting of existing *vasa vasorum* endothelial cells are supported structurally and via paracrine regulation by pericytes, fibroblasts and haematopoietic cells, especially macrophages^7,25–27^. On the other, adventitial EPCs may provide a local ancestral source of endothelial cells for postnatal vasculogenesis^5,6^. Having already discovered that mouse arteries contain AMPCs that are Sca-1^+^CD45^+^^13^, we embarked on this study in the expectation that this population would also have pro-angiogenic capacity to support adventitial neovascularisation. Our findings as summarised below, were both anticipated and unanticipated.

We first confirmed that in C57BL/6 mouse aorta, the adventitial Sca-1^+^ compartment is heterogeneous. As expected in the normal adventitial environment, the Sca-1^+^CD45^+^ subpopulation displayed negligible expression of mature endothelial surface markers, with most of these antigens present on the numerically more abundant Sca-1^+^CD45^−^ cells, that were already known to have endothelial plasticity^5,15^. Despite this, adventitial Sca-1^+^CD45^+^ cells were found to possess striking angiogenic and vasculogenic capacity across a range of *in vitro*, *in vivo*, and gene profiling studies. This exceeded that of adventitial Sca-1^−^CD45^+^ leukocytes when tested in Matrigel cord-forming assays and the hindlimb ischaemia model. Although we had speculated that the angiogenic properties of the Sca-1^+^CD45^+^ subpopulation would be mediated indirectly through formation of paracrine supportive macrophages, the results of our study support *bona fide* endothelial plasticity and vasculogenesis. Sca-1^+^CD45^+^ cells transformed *in vitro* into a CD31^+^ state specifically under endothelial inductive, but not other culture conditions. Most remarkably they also generated intact vWF^+^ and CD31^+^ microvessels, as well as LYVE1^+^ lymphatic vessels *in vivo*, with complementary evidence for direct contributions to adventitial and peri-adventitial microvessels in the carotid transfer experiment, and functional tissue perfusion in the hindlimb model of ischaemia. Pending further work, these results provide explanation for our other observation that Sca-1 and CD45 were co-expressed on the endothelium of adventitial and plaque microvessels in atherosclerotic *ApoE*^−/−^ mouse aortas and suggest the involvement of Sca-1^+^CD45^+^ cells in atherosclerotic *vasa vasorum* formation.

Since their original discovery from BM and peripheral blood^28^, the definition, cell lineage and hierarchy of EPCs have remained controversial^29^. Myeloid cells, mesenchymal stem cells and other cell types that are capable of promoting angiogenesis through indirect mechanisms rather than true endothelial differentiation, have notoriously been mislabelled as EPCs, despite being unable to form endothelial layers of vessels *de novo*. Within the existing literature about vascular-resident progenitor cells, Ingram *et al*. were the first to identify a clonogenic hierarchy of EPCs from isolates of human vascular endothelial cells, without localising the distribution of these cells to a mural layer of the vessel wall^30^. Subsequently Zengin and co-workers located a CD34^+^ progenitor population in the inner adventitial lining of human internal thoracic arteries and inferred the contribution of these cells to capillary-like sprouts from *ex vivo* ring assays that expressed endothelial markers and were surrounded by CD45^+^ cells^6^. Importantly, they noted that it was not possible to determine the exact source or fate of these adventitial CD45^+^ cells and found no evidence that CD45^+^ cells participated in capillary formation or co-expressed endothelial antigens. This is a key point of difference from the current study, where we directly compared different subpopulations of adventitial cells, and elucidated marked differences between the vasculogenic capacity and endothelial fate of the Sca-1^+^ (known to contain AMPCs) and Sca-1^−^ fractions (known to have a higher content of monocytes/macrophages but no AMPCs^13^) of the CD45^+^ compartment.

Stem cell antigen-1 is best known as an identifying marker of BM haematopoietic stem cells and different types of tissue-resident stem/progenitor cells in mice^31^. We and others have reported the abundance of Sca-1 expression in the adventitia of postnatal mouse arteries, where it has been used to identify and isolate vascular wall-resident stem cells^11–13,15,16,32,33^. Other groups have ascribed plasticity for endothelial, smooth muscle and mesenchymal progeny, including fibroblasts, to murine adventitial Sca-1^+^CD45^−^ cells^15–17,33,34^ and to corresponding adventitial progenitor cells in human vasculature^35,36^. Lineage-tracing has revealed that adventitial Sca-1^+^CD45^−^ cells do not originate from BM^5,15,32^, nor do they possess any haematopoietic potential^13^. Recently Majesky *et al*. used *in vivo* fate-mapping approaches, including tamoxifen-inducible *Myh11*-CreER^T2^ transgenic mice, combined with a smooth muscle cell epigenetic lineage mark, to show that 30–60% of adventitial Sca-1^+^ cells are derived from differentiated smooth muscle cells^33^. Almost 99% of these cells were Sca-1^+^CD45^−^, with the vast majority of adventitial Sca-1^+^CD45^+^ cells therefore originating from an alternative source.

Sca-1 has also been found on mature endothelial cells, which may themselves display endothelial plasticity in disease^5,31,37^. Patel *et al*. recently identified a novel endothelial hierarchy present in different postnatal mouse tissues, including normal mouse aorta^5^. Three subpopulations of endothelial cells were identified among VE-Cadherin^+^CD45^−^ cells, comprising CD31^−/Lo^VEGFR2^Lo/intracellular^ endovascular progenitors (EVPs), CD31^int^VEGFR2^Lo/intracellular^ transit amplifying precursors and definitive differentiated CD31^Hi^VEGFR2^Hi/extracellular^ endothelial cells. Notably, each of these was identified only after excluding the CD45^+^ fraction, and each expressed Sca-1 (i.e. Sca-1^+^CD45^−^). In keeping with this and flow cytometry analysis reported in the Majesky study^33^, we also observed that mature endothelial markers were exclusively expressed on the CD45^−^ subset of cells in non-atherosclerotic C57BL/6 aortas. However, this was not the case in atherosclerotic aortas from *ApoE*^−/−^ mice, nor in the adventitial vascular sprouts induced during *ex vivo* aortic ring assays from C57BL/6 mice. In both instances we found a surprisingly high level of Sca-1^+^CD45^+^ co-expression with a range of endothelial markers, including CD31, CD144, VEGFR2 and TIE2.

In the study by Patel, the self-renewing, clonal capacity of EVPs was established in a Matrigel-based assay^5^, whereby EVPs from mouse aorta or tumour microenvironment required at least three days to form endothelial colonies, before producing branching outgrowths which increased in length after day 4 through to day 7. We observed a similar time-frame of *de novo* cord formation from both Sca-1^+^CD45^+^ and Sca-1^+^CD45^−^ adventitial cells in Matrigel, although there was a trend for greater capacity to do this for Sca-1^+^CD45^+^ cells. Our study demonstrates the ability of aortic Sca-1^+^CD45^+^ progenitors to differentiate away from the CD45^+^ myeloid lineage in the process of forming endothelial cells under vasculogenic conditions. Moreover, with loss of CD45 expression, the Sca-1^+^CD45^+^ fraction gave rise to a high percentage of Sca-1^+^CD45^−^ cells in Matrigel, whereas the reverse did not occur. Interestingly, many of the angiogenic/vasculogenic genes found to be expressed highly in Sca-1^+^CD45^+^ cells were also among those that were upregulated in EVPs compared to mature endothelial cells in the Patel study^5^: *Mmp2*, *Mmp3*, *Il33*, *Sox9*, *Cd34, Egfr*. Therefore it is evident that both the CD45^+^ and CD45^−^ subsets of the adventitial Sca-1^+^ population have potential to produce endothelial-fated progeny and participate in vasculogenesis. Questions now remain as to how these two subpopulations inter-relate developmentally, with our findings suggesting for the first time that Sca-1^+^CD45^+^ progenitors may be hierarchically ancestral to at least a subpopulation of Sca-1^+^CD45^−^ cells present in murine adventitia.

In combination with our previous results^13^, this study has revealed the bipotent nature of the adventitial Sca-1^+^CD45^+^ population, with capacity to produce both myeloid (macrophage) and endothelial progeny. Although we did not add macrophage-specific growth factors to the Matrigel cord-forming assay, Sca-1^+^CD45^+^ cells still produced a small percentage of macrophages which selectively clustered at branch points. It followed that their depletion by clodronate showed a strong trend toward reducing cord branching, indicating that the macrophage progeny of Sca-1^+^CD45^+^ progenitors acted as vascular fusion cells to support endothelial tip cell anastomosis, as has been described by others^18^. Although our carotid and hindlimb adoptive transfer experiments demonstrated the ability of Sca-1^+^CD45^+^ cells to produce endothelial-lined neovessels *in vivo*, it should be noted that we did not quantify the proportion of donor cells that were able to do this nor their relative predisposition to producing macrophages versus endothelium.

The lack of a significant increase in capillary or arteriolar density 14 days after Sca-1^+^CD45^+^ cells were injected into ischaemic hindlimbs, was at odds with the significant augmentation in perfusion ratio that these cells achieved compared to cell-free control. There are several possible reasons for this discrepancy. Firstly, C57BL/6 mice have an inherent ability to quickly undergo spontaneous angiogenesis-mediated recovery after artery ligation, even in the absence of treatment, which may make it difficult to detect subtle differences in capillary density. Secondly, other mechanisms in addition to angiogenesis and arteriogenesis are involved in the reperfusion response to hindlimb ischaemia. These include skeletal muscle regeneration and necrotic tissue clearance which were not measured here. Finally, although we quantified capillary and arteriolar density from three randomly selected 5 µm-thick sections of gastrocnemius muscle obtained from each animal, these measures may be vulnerable to a degree of sampling bias.

It is also important to emphasise that like other studies that have reported on the multipotency of different adventitial progenitor cells^15,33,35,36^, our findings still relate to population biology, and do not prove multipotency at the level of individual Sca-1^+^CD45^+^ cells. We therefore cannot yet claim to have proven the presence of haemangioblasts in postnatal vasculature. The notion that such cells exist was introduced in the 1930s, borne out of the intimate relationship between developmental vasculogenesis and primitive haematopoiesis in extraembryonic yolk sac^38^. Yolk sac erythromyeloid progenitor cells are now well known to give rise to self-renewing macrophages that reside in different tissues, including those found in murine adventitia especially in early postnatal life^39^. Most recently, they have also been shown to generate vascular endothelium in both developing and postnatal mouse brain^40^. We have already determined that the AMPC subset of adventitial Sca-1^+^CD45^+^ cells are not derived from BM or splenic haematopoiesis^13^. Although we did not exclude a BM source for the vasculogenic Sca-1^+^CD45^+^ progenitor cells described here, results from studies of other types of vascular wall EPCs suggest that this is unlikely^5^.

Directions for future research are therefore to determine at a clonal, single cell level whether postnatal adventitial Sca-1^+^CD45^+^ progenitors contain *bona fide* haemangioblasts, and if so, to elucidate their embryonic origins. Other objectives will be to determine whether the vasculogenic properties of these cells are adaptive or maladaptive in diseases such as atherosclerosis; whether similar cells are present in human arteries, where Sca-1 cannot be used as a candidate marker for their identification; and whether there are corresponding populations of CD45^+^ progenitors residing around the microvasculature of other tissues, that might participate in vasculogenic responses to wound healing, ischaemia and cancer.

In conclusion, we have identified that in addition to serving as a local source of macrophage expansion in the mouse vasculature, adventitial Sca-1^+^CD45^+^ progenitor cells are also vasculogenic, with relevance to the origins of *vasa vasorum* formation in atherosclerosis.

## Methods

### Mice

Breeding pairs of C57BL/6 (C57BL/6 J), *ApoE*^−/−^ (B6.129 P2-*ApoE*^*tm1Unc*^/J), GFP (C57BL/6-Tg(UBC-GFP)30Scha/J), Ly6a-GFP transgenic (B6.Cg-Tg(Ly6a-EGFP)G5Dzk/J) and *Cx3cr1*^GFP/+^ (B6.129P-*Cx3cr1*^*tm1Litt*^/J) mouse strains were acquired from The Jackson Laboratory (Bar Harbor, ME, USA). Mice were housed in the animal care facilities at Mayo Clinic and the South Australian Health and Medical Research Institute (SAHMRI), and were maintained on standard chow diet or atherogenic diet (Teklad diet #88137 [42% caloric intake from fat], Harlan Laboratories, Madison, WI, USA), as specified. Both males and females were used at ages specified throughout the text and figure legends. All animal experiments were performed in accordance with the standards stated in the Guide for the Care and Use of Laboratory Animals (Institute of Laboratory Animal Resources, National Academy of Sciences, Bethesda, MD, USA) and were approved by the Mayo Clinic and SAHMRI Institutional Animal Care and Use Committees.

### Preparation of single cell suspensions

Preparation of cell disaggregates from aortic adventitia was performed from mice, as described previously^12^. Aortas were dissected out intact, along their entire length from aortic valve to iliac bifurcation and flushed extensively with heparinised phosphate buffered saline (PBS), before microscopic dissection of surrounding perivascular fat and mechanical separation of the adventitia. Adventitial pieces were then incubated for up to 2 hours at 37 °C in a solution containing Liberase TM (50 μg/ml) (Roche Applied Science, Mannheim, Germany). Disaggregates were then neutralised in serum-replete media and washed, before performing cell counts.

Freshly isolated, single cell adventitial disaggregates were subjected to multi-column magnetic activated cell sorting (MACS), as per manufacturer recommendations (Miltenyi Biotec Inc, Auburn, CA, USA), to obtain four subpopulations with differential expression of Sca-1 and CD45. For each separation experiment, aortic adventitia from 6–10 12w mice were pooled.

In the Matrigel-based aortic ring and vascular cord-forming assays, cells were retrieved from wells by scraping the Matrigel and then digested in type IV collagenase (0.2 mg/mL in PBS) (Sigma-Aldrich Inc., St Louis, MO, USA) for 45 minutes. After neutralisation with Iscove’s Modified Dulbecco’s Medium (Sigma-Aldrich) supplemented with 10% foetal calf serum (FCS) (Cell Sera, NSW, Australia), single cell suspensions were then immunostained for flow cytometry as described below.

### Flow cytometry

Single cell suspensions were resuspended in aliquots of ≤ 10^6^ cells in 100 μL MACS buffer. After blocking for 15 minutes at 4 °C, cells were incubated for 45 minutes with fluorochrome-conjugated, anti-mouse monoclonal antibodies to different cell surface markers that are catalogued in Supplementary Table 2. Fluorescence minus one (FMO) or appropriate isotype matched negative controls were used to set thresholds for the expression of different markers. All antibodies were purchased from BioLegend (San Diego, CA, USA) and BD Biosciences (San Jose, CA, USA). Samples were then washed and fixed in formalin/PBS for analysis with an BD LSRFortessa^TM^ X-20 Flow Cytometer System (BD Biosciences, San Jose, CA, USA). Data files were analysed using FlowJo V.10.0.8 LLC software (Tree Star Inc., Ashland, OR, USA).

### Tissue immunofluorescent staining and confocal microscopy

Intact tissue samples were embedded in Optimal Cutting Temperature (O.C.T.) compound (Sakura Finetek USA, Inc., Torrance, CA, USA). Five µm thick frozen sections were cut, fixed and blocked with either 10% normal goat or donkey serum, before incubating with primary and then secondary antibodies that are detailed in Supplementary Table 3. Nuclei were stained with Hoechst or DAPI (Sigma Aldrich). For the purpose of detecting GFP^+^ chimerism in cell transfer studies, different tissue extracts (carotid arteries, gastrocnemius) were fixed overnight in 10% formalin and then embedded in O.C.T. Chimerism (cell retention) was determined by a combination of detecting endogenous EGFP expression and GFP labelling with either a rabbit polyclonal or rat monoclonal antibody. Microscopy was performed with a Zeiss LSM 510 laser scanning confocal microscope system (Carl Zeiss BmbH, Germany).

### RNA microarray and RT-qPCR validation

For the purpose of RNA isolation for microarray and RT-qPCR, aortas from six 12w C57BL/6 mice per donor experiment were pooled to allow MACS-based separation of the Sca-1^+^CD45^+^, Sca-1^−^CD45^+^ and Sca-1^+^CD45^−^ adventitial fractions. Total RNA was isolated and purified immediately using an RNeasy^®^ Plus Mini Kit (Qiagen, Germany), as per manufacturer’s instructions.

RNA microarray was performed to compare the transcriptional profiles of adventitial Sca-1^+^CD45^+^ and Sca-1^−^CD45^+^ cells from three donor experiments. The quality of all total RNA samples was assessed according to manufacturer’s instructions for the Agilent 2100 Bioanalyzer (Santa Clara, CA, USA). High-quality samples were labelled according to instructions for the Illumina Direct Hyb labelling method (San Diego, CA, USA). Briefly, 100 ng of total RNA was reverse transcribed with T7 Oligo d(T) to create second strand cDNA in concordance with the protocol for the Illumina TotalPrep RNA Amplification kit (Ambion/Life Technologies, Foster City, CA). Subsequently, the products were column purified (Ambion/Life Technologies) and then *in vitro* transcribed to generate biotin-labeled cRNA. cRNA products were column purified and hybridised onto Illumina MouseWG-6 Beadchips for 16 hours at 58 °C. Following hybridisation, the arrays were washed, stained with streptavidin-cy3 conjugate, and then scanned in an Illumina BeadArray Reader. All quality assessment parameters were determined to be within normal ranges before proceeding to the final data reduction.

The log-2 of the gene expression data was normalised using the non-linear normalisation *fastlo*^41^. To assess differential expression between groups of interest, the LIMMA package in R^42^ was utilised to implement the empirical Bayes method of Smyth^43^ to shrink the gene-wise sample variances towards a common value. The false discovery rate (FDR^44^), which is the expected proportion of false discoveries amongst the rejected hypotheses, was also calculated.

Pathway analysis by Ingenuity IPA (http://www.ingenuity.com) was conducted to identify significantly enriched canonical pathways, functional groups or biological processes, with focus for this study on genes involved in angiogenesis or vasculogenesis. Selected angiogenesis/vasculogenesis-related genes that were differentially regulated between Sca-1^+^CD45^+^ and Sca-1^−^CD45^+^ cells and had an FDR <0.05 from microarray were validated using hydrolysis (Taqman) probe-based RT-qPCR (MIQE reference - http://miqe.gene-quantification.info/). All assays and reagents were ordered from Life Technologies (Foster City, CA, USA) (Supplementary Table 4). Cq values were calculated using the Viia7 software for individual qPCR run. The data were imported and analysed in the Expression Suite Software (Life Technologies) and the reported delta-delta Ct was plotted as log-fold difference.

### *In vitro* culture-based differentiation

Freshly isolated C57BL/6 aortic adventitial cells were fractionated into different Sca-1/CD45 subpopulations which were then seeded in collagen-coated glass chamber slides (Nalge Nunc International, Naperville, IL) at 2 × 10^4^ cells in 200 μL in one of four different media conditions: (1) RPMI-1640 (Sigma-Aldrich) supplemented with 10% FCS (RPMI-10); (2) RPMI-10 with 20 ng/mL M-CSF (PeproTech Inc., Rocky Hill, NJ); (3) RPMI-10 with 20 ng/mL IL-4 and 20 ng/mL GM-CSF; (4) endothelial growth medium (Lonza, Walkersville, MD) supplemented with 10% FCS (EGM-10). Medium was changed every three days until day 10, at which time the wells were fixed with cold methanol and permeated with 0.1% triton before immunostaining (as described above) to detect endothelial (Isolectin, CD31) markers, with Hoechst nuclear counter-staining.

### Aortic ring outgrowth model

To evaluate the participation of adventitial cells in local angiogenic processes, aortic ring studies were performed, as previously described^45^. Aortas were harvested from 12w Ly6a-GFP or C57BL/6 mice. Aortic explants were carefully flushed to remove blood and dissected free of surrounding adipose. Some aortas were then used with all three mural layers intact, while others had their intima denuded by mechanical needle injury and/or their adventitia completely dissected away. Aortic rings of 1 mm thickness were then cut, embedded in Matrigel and overlaid with EGM containing 5% FCS and 20 ng/mL vascular endothelial growth factor (VEGF), platelet-derived growth factor (PDGF) and fibroblast growth factor (FGF) (PeproTech Inc.). Medium was changed after two days and aortic sprouts were imaged until day 7. Aortic rings from Ly6a-GFP mice were then fixed with formalin before the Matrigel was removed intact and embedded in OCT for sectioning and immunofluorescent staining. Ring assays from C57BL/6 mice were processed as described above to retrieve their cellular content and prepare single cell suspensions for flow cytometry.

### Matrigel-based vascular cord formation

Freshly isolated murine adventitial Sca-1/CD45 cell fractions were suspended in EGM-10 and plated on growth factor–reduced Matrigel (BD Biosciences, San Jose, CA) at 3 × 10^4^ cells per well in 200 µL volume, to compare their intrinsic capacity to form vascular-like cords over time. Each cell subset was assayed in either triplicate or quadruplicate per experiment (n ≥ 3 independent experiments performed for each cell type). From 24 hours onward, wells were inspected daily and photographed under light microscopy until day 7 to detect the presence of cords, identified as cellular extensions linking cell masses or branch points. In some experiments, clodronate or PBS control liposomes (clodronateliposomes.org, Vrije Universiteit, Netherlands) were added to wells at a concentration of 0.45 mg/mL alternate daily from day 3 until day 7. Images were captured at x40 magnification in five different regions (upper centre, centre, lower centre, left centre, right centre) to encompass the entire well. Cords were traced *post-hoc* and total cord length quantified from each image and in turn each well using MetaMorph microscopy automation and image analysis software (Molecular Devices, LLC, Sunnyvale, CA).

In addition, co-culture experiments were performed in which the different adventitial Sca-1/CD45 subpopulations from ubiquitous GFP donor mice were plated with *ex vivo* cultured HUVECs (ATCC, Manassas, VA) that had been labelled with CellTracker Orange (Invitrogen, Eugene, OR) at 1:1 density (1.5 × 10^4^ per cell type per well). These wells were imaged by light microscopy and confocal microscopy (488 nm filter for EGFP excitation and 594 nm for CellTracker Orange at 594 nm) at different time points during five days of culture.

### Hindlimb ischaemia model

The murine hindlimb ischaemia model was performed as described previously, using six month-old male C57BL/6 mice^46^. Briefly, the proximal portion of the femoral artery and the distal saphenous artery were both suture ligated, as well as the popliteal artery and all branches in between, and an arterectomy performed to remove the intermediate segment of vessel. Blood flow through the ischaemic (left) and nonischaemic (right) limbs was immediately determined and compared to baseline using laser Doppler perfusion imaging (PeriScan PIM 3 System, Perimed AB, Stockholm, Sweden). Once adequate ischaemia was established, the surgical site was closed. Aortic adventitial cells, freshly fractionated into Sca-1/CD45 subpopulations from 12w GFP donor mice (7.5 × 10^5^ in 60 μL Matrigel), were then administered as a single intramuscular injection (27 G needle) into the gastrocnemius muscle of the ischaemic limb. One group of mice received Matrigel only, as a cell-free control.

Mice were assessed clinically before and after surgery (at days 0, 7 and 14), using well-established clinical scoring (0 = normal gait movement, 1 = pale foot colouring/gait abnormality, 2 = ischaemic tissues in less than half the foot without lower limb necrosis, 3 = ischaemic tissues in less than half the foot with lower limb necrosis, 4 = ischaemic tissues in more than half the foot, 5 = loss of half the lower limb) and foot movement (0 = normal plantar/toe flexion on tail traction, 1 = no toe flexion, 2 = no plantar flexion, 3 = dragging of foot) scales. Laser Doppler perfusion scans were repeated at day 7 and 14 under general anaesthesia to determine the perfusion ratio between the ischaemic and non-ischaemic limb in mice that had received Matrigel control, aortic Sca-1^+^CD45^+^ or Sca-1^−^CD45^+^ cells (n = 5–6 per group). Euthanasia was performed after the final scan on day 14, and the gastrocnemius muscle of both limbs was harvested for immunofluorescent detection of GFP^+^ cell retention and fate.

### Carotid adventitial delivery

Aortic adventitial cells from 12w GFP donor mice were freshly isolated and MACS separated into the four Sca-1/CD45 fractions. Cells were resuspended in Matrigel (5 × 10^5^ in 20 μL) and injected (27 G needle) into the adventitia of the left carotid artery of recipient 8w *ApoE*^−/−^ mice, as described previously^13^. Mice were commenced on atherogenic diet immediately after surgery, which was maintained until euthanasia 16 weeks later. At study completion, both carotid arteries were harvested, fixed and processed to investigate the fate of donor GFP^+^ cells.

### Statistical Analysis

Data-sets were tested for normality of distribution by D’Agostino-Pearson omnibus test. Statistical comparisons were performed with parametric or non-parametric unpaired or paired two-sample *t*-tests or ANOVA (with post-test comparisons), as specified. Results are expressed as mean ± standard deviation (sd) of multiple experiments, unless otherwise specified. In all cases, statistical significance was established at two-tailed *P* < 0.05.

### Accession Numbers

The Illumina Microarray data reported in this publication have been deposited in NCBI’s Gene Expression Omnibus and are accessible through the GEO Series accession number GSE127852 (https://www.ncbi.nlm.nih.gov/geo/query/acc.cgi?acc = GSE127852).

## Supplementary information

Supplemental File

## Data Availability

The datasets generated during and/or analysed during the current study are available from the corresponding author on reasonable request.
