# Supplementary material for: Vasculogenic properties of adventitial Sca-1+CD45+ progenitor cells in mice: a potential source of vasa vasorum in atherosclerosis
Source: Sci Rep. 2019 May 13;9:7286. doi: 10.1038/s41598-019-43765-8 (PMC6513996; doi:10.1038/s41598-019-43765-8)
Supplement: Supplementary file 1 — Supplemental File [file 41598_2019_43765_MOESM1_ESM.pdf]

Vasculogenic properties of adventitial Sca-1$^+$CD45$^+$ progenitor cells in mice: a potential source of vasa vasorum in atherosclerosis

Deborah Toledo-Flores[1#], Anna Williamson[1,2#], Nisha Schwarz[1], Sanuja Fernando[1,2], Catherine Dimasi[1], Tyra A. Witt[3], Thao M. Nguyen[1,2], Amrutesh S. Puranik[3], Colin D. Chue[3], Sinny Delacroix[2,3], Daniel B. Spoon[3], Claudine S. Bonder[2,4], Christina A. Bursill[1,2], Belinda A. Di Bartolo[1,2], Stephen J. Nicholls[1,2], Robert D. Simari[3,5], Peter J. Psaltis[1,2,3] *

[1] Vascular Research Centre, Heart Health Theme, South Australian Health and Medical Research Institute, Adelaide, Australia

[2] Adelaide Medical School, University of Adelaide, Adelaide, Australia

[3] Division of Cardiovascular Diseases, Mayo Clinic, Rochester, MN, USA

[4] Centre for Cancer Biology, University of South Australia & SA Pathology, Adelaide, Australia

[5] University of Kansas School of Medicine, Kansas City, KS, USA

# Co-first authors

* Address for Correspondence

Peter J. Psaltis, MBBS PhD

Vascular Research Centre, Heart Health Theme, Level 6

South Australian Health and Medical Research Institute, North Terrace, Adelaide,

South Australia, Australia, 5000

peter.psaltis@sahmri.com

Supplementary Figure Legends

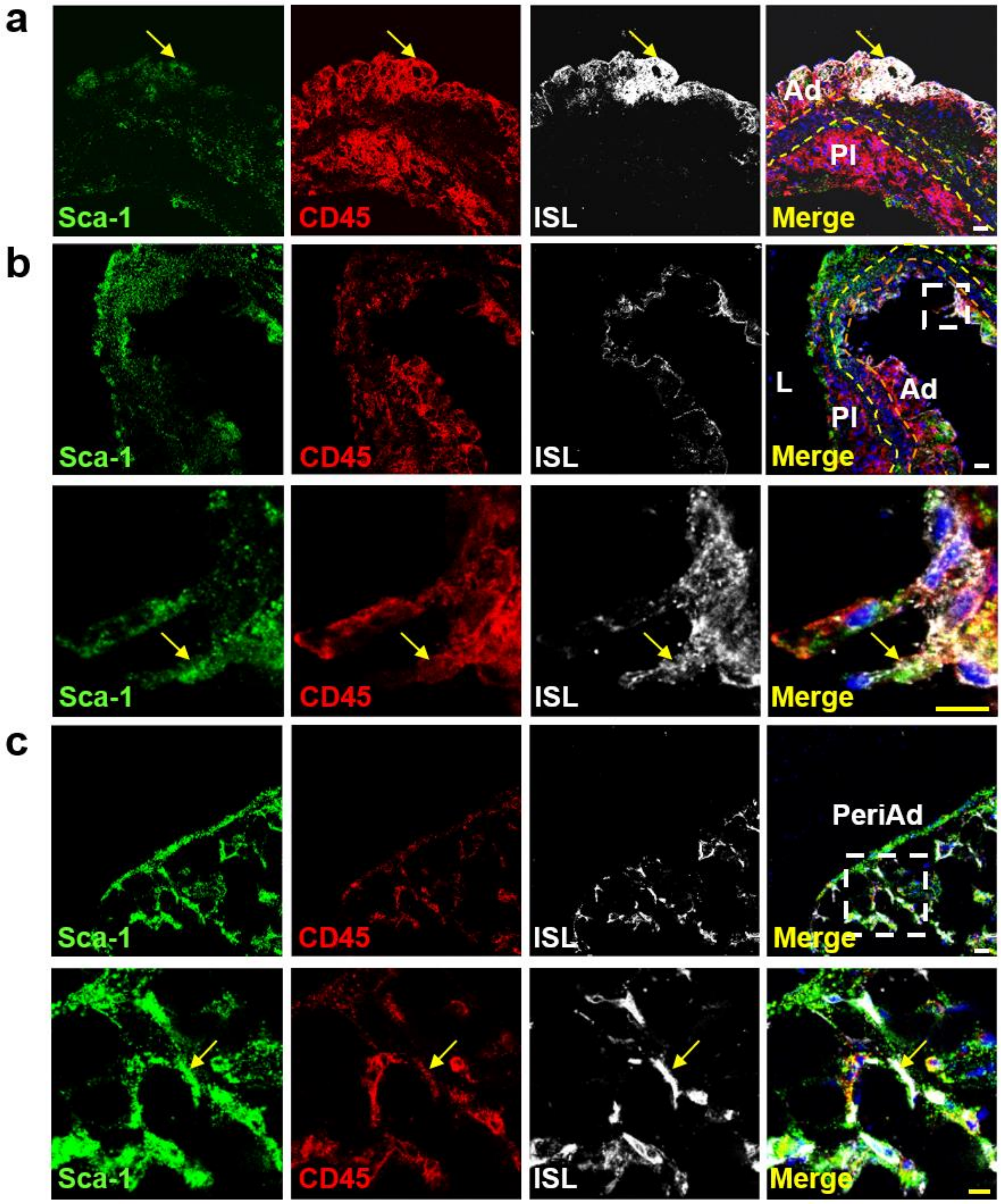

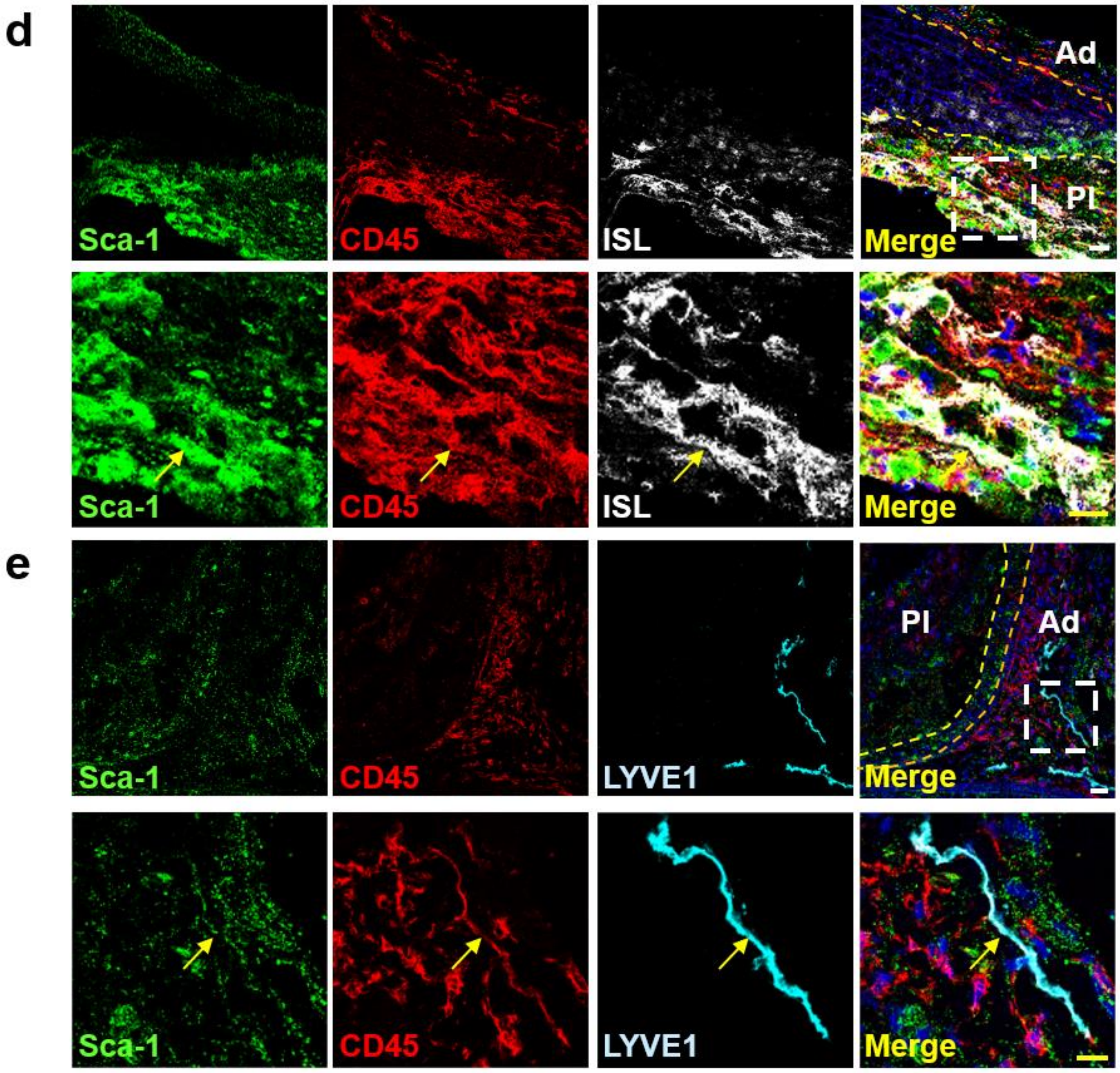

Supplementary Figure 1. Co-expression of Sca-1 and CD45 on microvessels in *ApoE*$^{-/-}$ aortic arch.

Immunofluorescent staining and confocal microscopy of different sections of aortic arch obtained from four different 24w *ApoE*$^{-/-}$ mice that had been maintained on atherogenic diet for 16w. **a-d**, Co-localisation of Sca-1 and CD45 with ISL^{+} microvessels in (a, b) adventitia, (c) peri-adventitial tissue and (d) atherosclerotic plaque. **e**, Co-staining of Sca-1 and CD45 with the lymphatic marker, LYVE1. The low magnification images in (a) correspond to **Figure 1e** in the main manuscript. In (b-e) the upper row shows lower magnification images, and the lower row shows the higher magnification images of the corresponding inset box. Examples of co-staining are indicated by yellow arrows. Nuclei are counterstained blue with

Hoechst. Internal and external elastic laminae are demarcated by broken yellow and orange lines respectively. Ad, adventitia; L, lumen; PeriAd, peri-adventitial tissue; Pl, plaque. Scale bars: 10µm (yellow), 20µm (white).

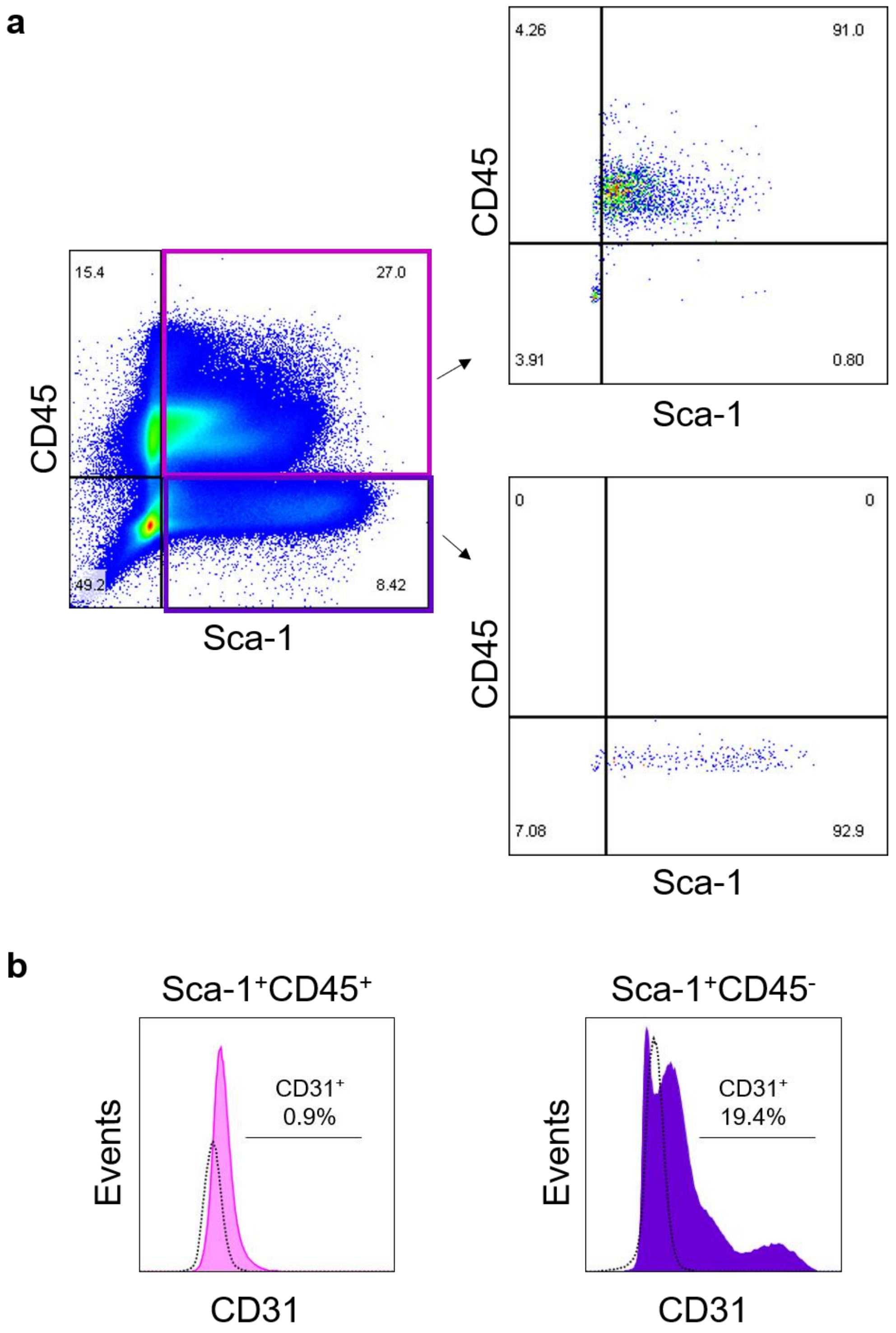

Supplementary Figure 2. Flow cytometry of freshly sorted aortic digests from C57BL/6 mice.

a, Representative flow cytometry dot plot is shown on the left for Sca-1 and CD45 from aortic cell digest pooled from n=10 12w C57BL/6 mice, immediately prior to sorting. To the

right are representative dot plots following sorting of the Sca-1$^+$CD45$^+$ (top right) and Sca-1$^+$CD45$^-$ (bottom right) subpopulations. **b,** Histograms are shown for CD31 expression in the freshly isolated Sca-1$^+$CD45$^+$ (pink) and Sca-1$^+$CD45$^-$ (blue) populations, with FMO control histogram shown as the underlying dotted line.

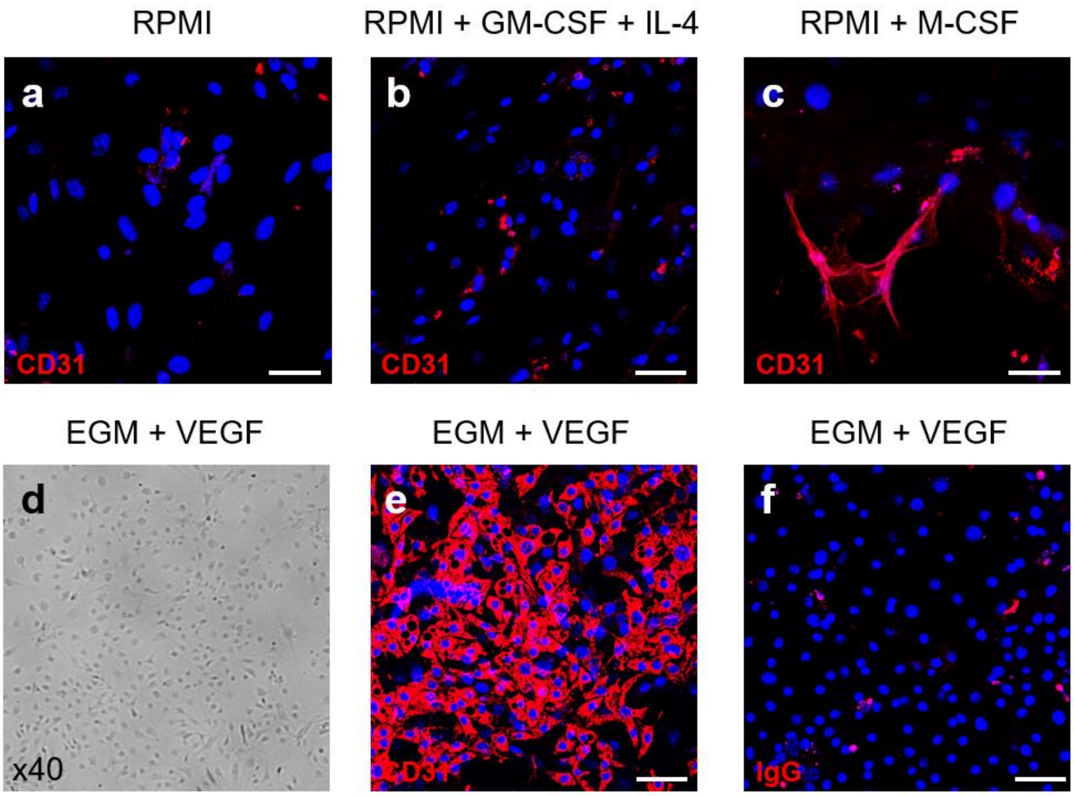

Supplementary Figure 3. Endothelial differentiation of adventitial Sca-1$^+$CD45$^+$ cells. Representative immunofluorescent staining for CD31 expression is shown for C57BL/6 adventitial Sca-1$^+$CD45$^+$ cells cultured for 10 days in media containing different growth factors. CD31 staining was negligible when cells were cultured in basal RPMI media (**a**) and RPMI supplemented with IL-4/GM-CSF (**b**), and modest in the presence of M-CSF (**c**). However, endothelial growth medium (EGM) supplemented with VEGF induced cobble-stone endothelial-like morphology on light microscopy (**d**) and uniform CD31 expression (**e**). IgG control staining for CD31 is shown in (**f**). Images are representative of n=2-3 separate experiments for each condition. Each experiment involved the pooling of aortas from 6 C57BL/6 mice. Nuclei are counterstained blue with Hoechst. Scale bar: 50µm (white).

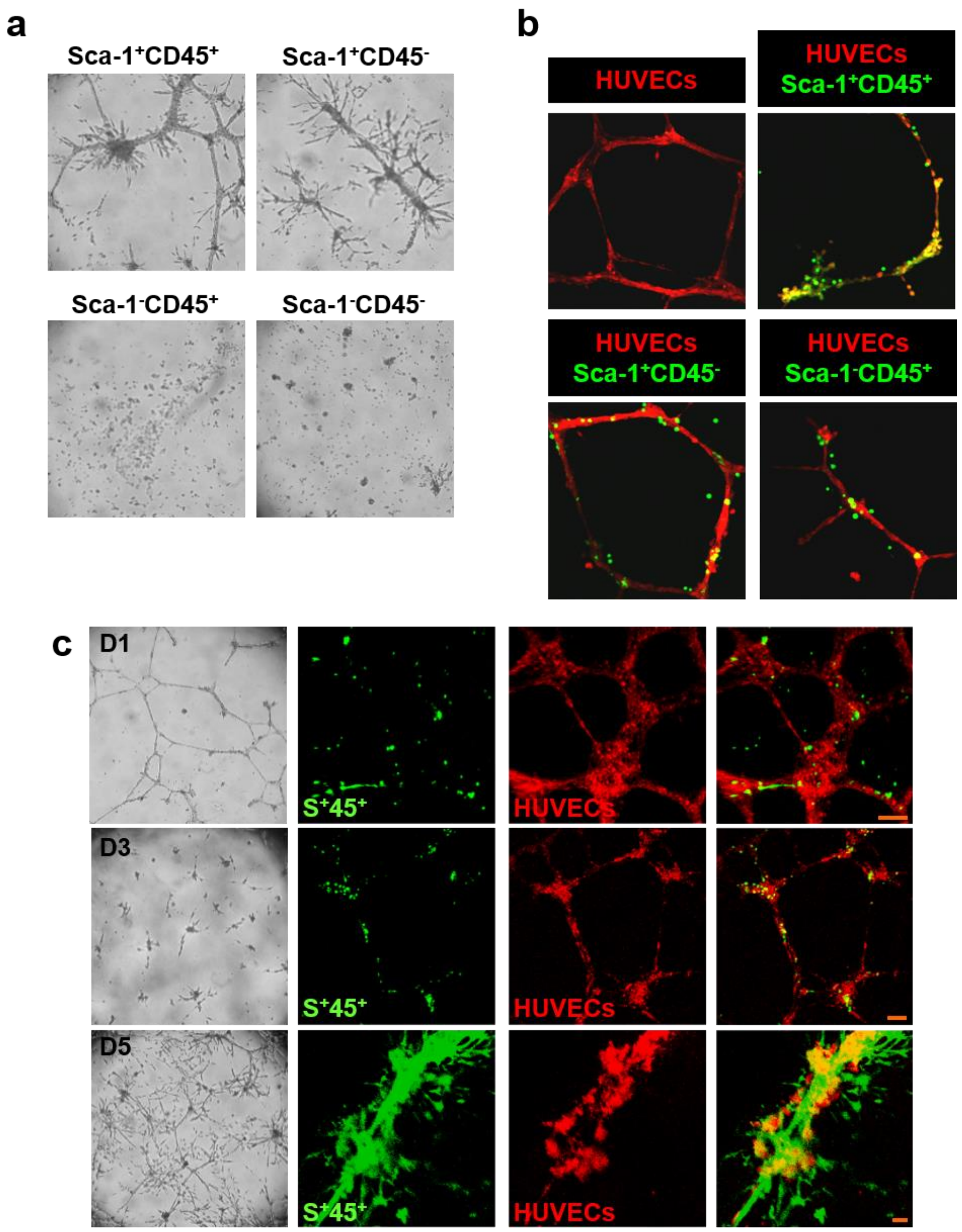

Supplementary Figure 4. Matrigel-based vascular cord-forming capacity of different adventitial Sca-1/CD45 subpopulations.

a, Light microscopic images (x40) taken on day 5 after culturing different adventitial Sca-1/CD45 subpopulations in Matrigel and endothelial growth medium. Note absence of cords from both Sca-1$^-$ populations compared to elaborate vascular-like networks formed from both Sca-1$^+$CD45$^+$ and Sca-1$^+$CD45$^-$ cells. **b**, Merged confocal images taken on day 3 after 1:1 co-culture of HUVECs (red) and different adventitial GFP$^+$ Sca-1/CD45 fractions (green) in

Matrigel. Sca-1$^+$CD45$^+$ cells integrated and contributed to cords to a greater extent than Sca-1$^+$CD45$^-$ and Sca-1$^-$CD45$^+$ cells. **c,** Time course of vascular cord formation after 1:1 co-culture of GFP$^+$Sca-1$^+$CD45$^+$ cells and HUVECs labelled with orange cell-tracker. D, day. Images are representative of n=2-4 independent experiments. Each experiment involved the pooling of aortas from n≥6 C57BL/6 donor mice. Scale bar: 50μm (orange).

Sca1$^+$CD45$^+$

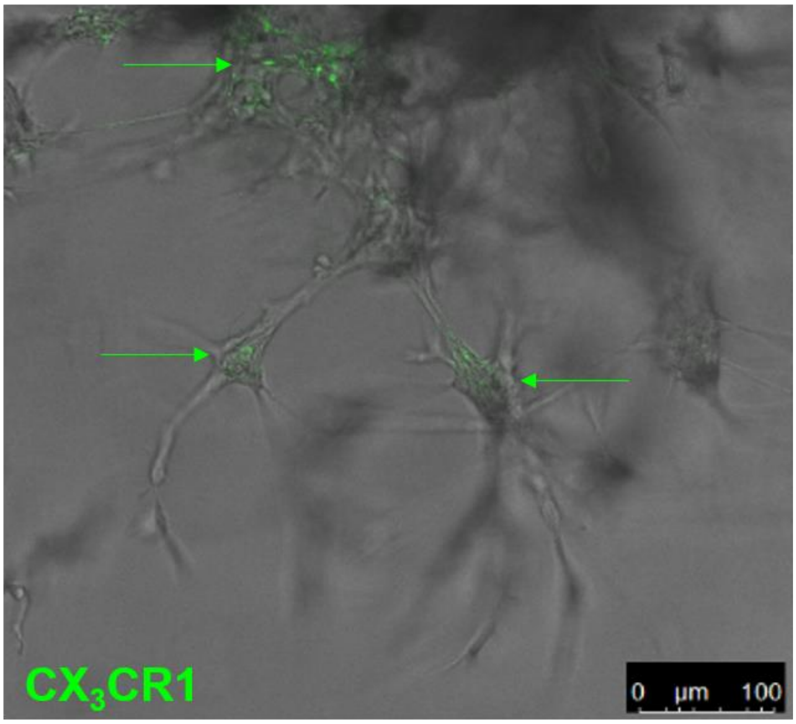

Sca1$^+$CD45$^-$

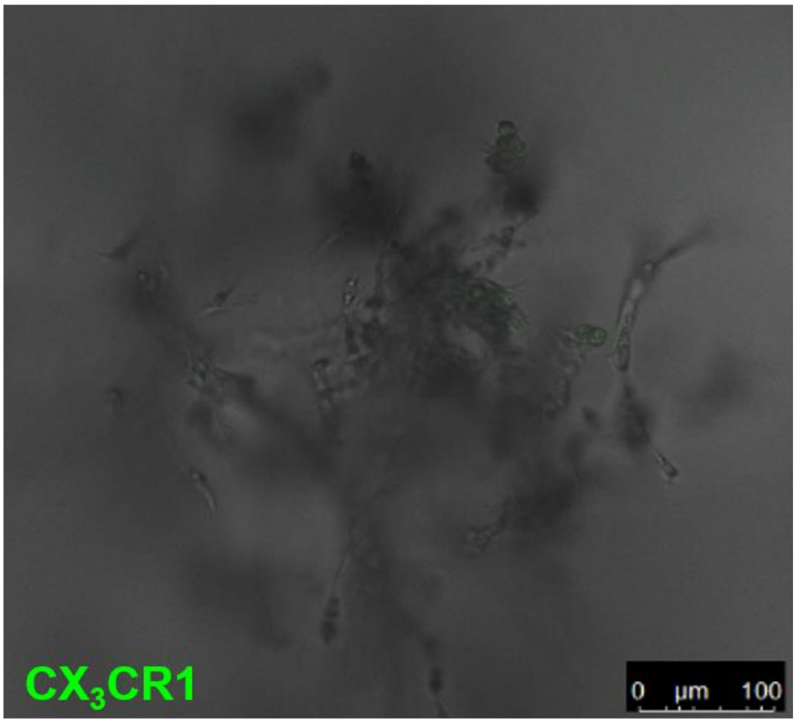

Supplementary Figure 5. Distribution of CX_3CR1^+ cells in vascular-like networks formed from adventitial Sca-1^+CD45^+ cells.

Merged images from phase contrast and immunofluorescent microscopy of cord networks from Sca-1^+CD45^+ (top) and Sca-1^+CD45^- (bottom) aortic cells sorted from *Cx3cr1*$^{GFP/+}$ mice. CX_3CR1^+ (GFP$^+$) cells, which include macrophages, were observed at the connecting intersections of cords grown from the Sca-1^+CD45^+ (green arrows) but not Sca-1^+CD45^- fraction.

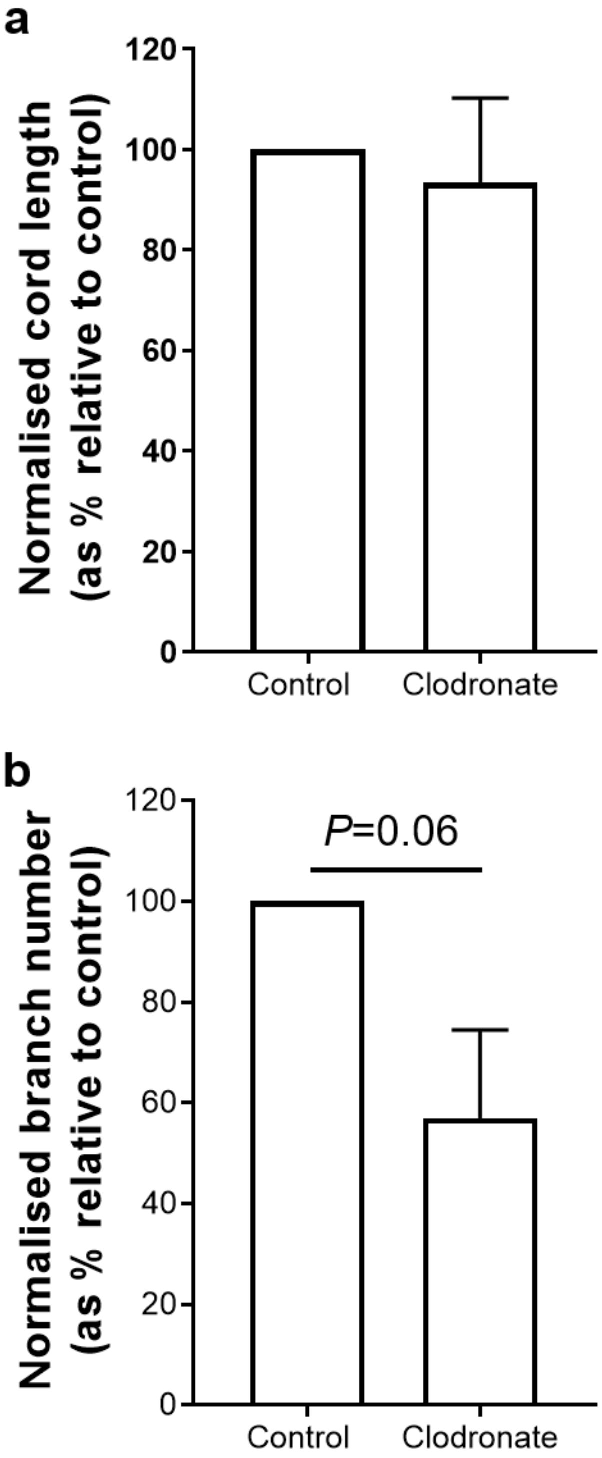

Supplementary Figure 6. Effect of clodronate on cord formation from adventitial Sca-1^{+}CD45^{+} cells.

Normalised data for (**a**) total cord length and (**b**) number of branch points after adventitial Sca-1^{+}CD45^{+} cells were cultured in Matrigel for seven days in the presence or absence of

clodronate liposomes which were used to deplete their macrophage progeny. Results are expressed as the mean±sd % relative to the no clodronate PBS control condition obtained from n=5 experiments. Analysis for this experiment was performed using ImageJ software (NIH, Bethesda, Maryland, USA). Statistical comparisons were performed by Wilcoxon matched-pairs signed rank test.

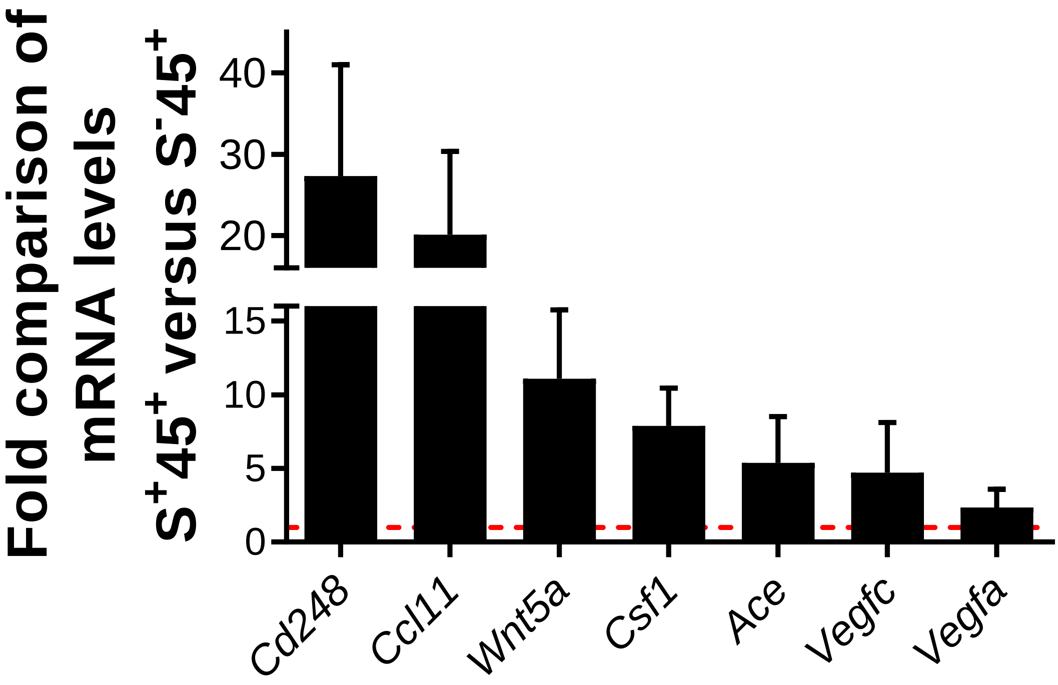

Supplementary Figure 7. mRNA levels of selected angiogenic/vasculogenic genes.

RT-qPCR validation was performed for seven genes that are known to be involved in angiogenesis or vasculogenesis and were found by unbiased microarray to be more highly expressed in adventitial Sca-1$^+$CD45$^+$ cells than Sca-1$^-$CD45$^+$ cells from 12w C57BL/6 aortas. Graph summarises the mean±sd fold comparison of mRNA expression between Sca-1$^+$CD45$^+$ and Sca-1$^-$CD45$^+$ cells, with the red dotted line corresponding to equivalence. N=3 different experiments. Each experiment involved the pooling of aortas from 6 C57BL/6 donor mice.

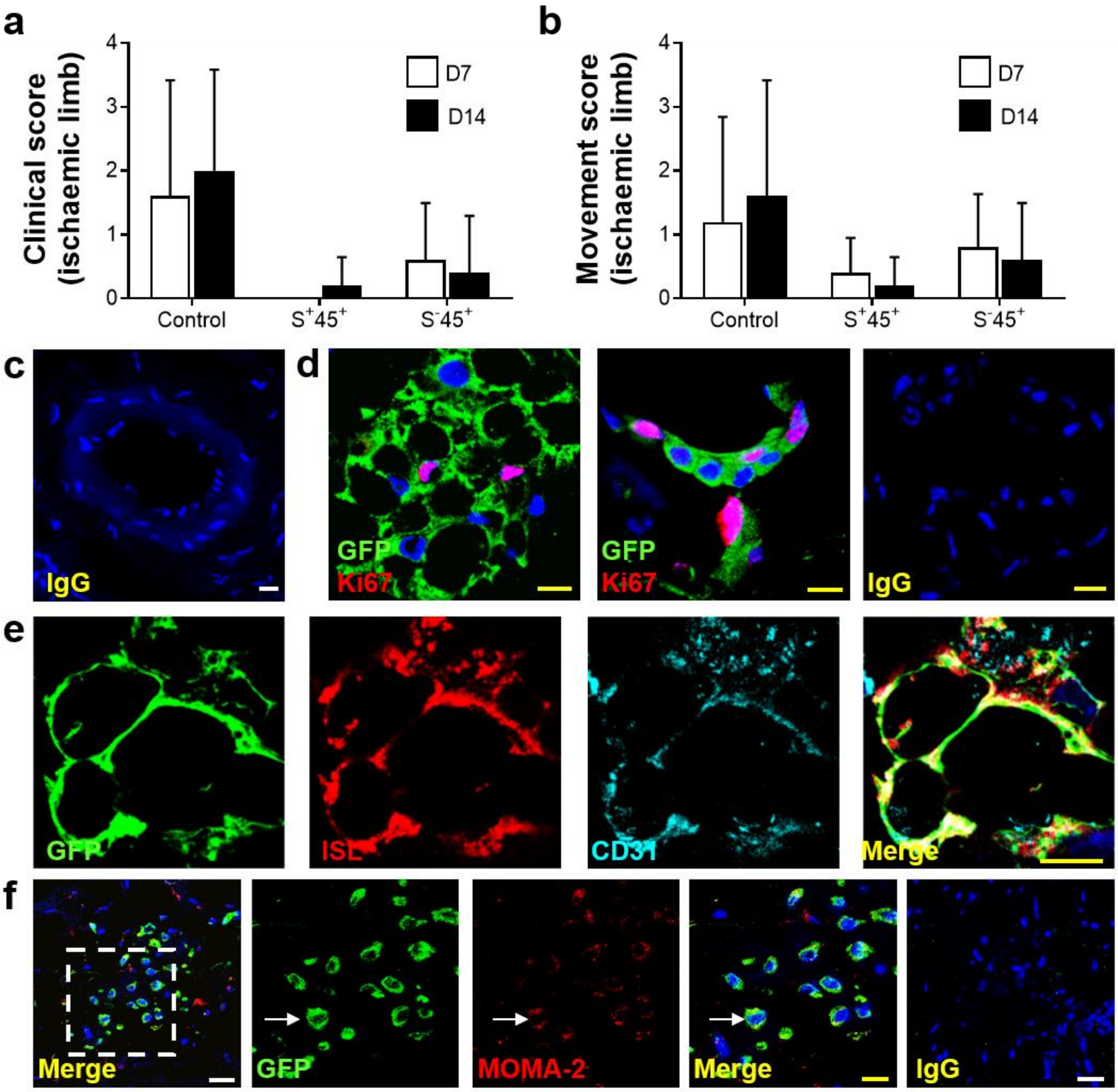

Supplementary Figure 8. Fate of adventitial Sca-1⁺CD45⁺ cells in hindlimb ischaemia.
a, Clinical and **b**, movement scores for the ischaemic hindlimb at day 7 and 14 after arterial ligation and injection of cell-free Matrigel (control) or freshly-isolated aortic adventitial Sca-1$^+$CD45$^+$ (S^+45^+) or Sca-1$^-$CD45$^+$ (S^-45^+) cells. Data are shown as mean±sd from n=5-6 per group. Despite trends favouring better clinical recovery after Sca-1$^+$CD45$^+$ transfer, there were no statistically significant differences between groups as per Kruskal-Wallis test. **c**, Merged image from IgG isotope control staining for GFP, CD31 and TER119 (please refer to **Figure 5c**). **d-f**, Co-staining for GFP and (d) the proliferative marker, Ki67 (different sections from two mice shown), (e) CD31 and binding to isolectin (ISL), and (f) the

macrophage marker, MOMA-2, from different sections of gastrocnemius muscle 14 days after ischaemic surgery and intramuscular injection of aortic $GFP^{+}Sca\text{-}1^{+}CD45^{+}$ cells. Higher magnification images in (f) correspond to the preceding inset box and arrow denotes $GFP^{+}MOMA\text{-}2^{+}$ cell. Merged images from the relevant IgG control antibodies are also shown where indicated. Nuclei are counterstained blue with Hoechst. Scale bars: 10µm (yellow), 20µm (white).

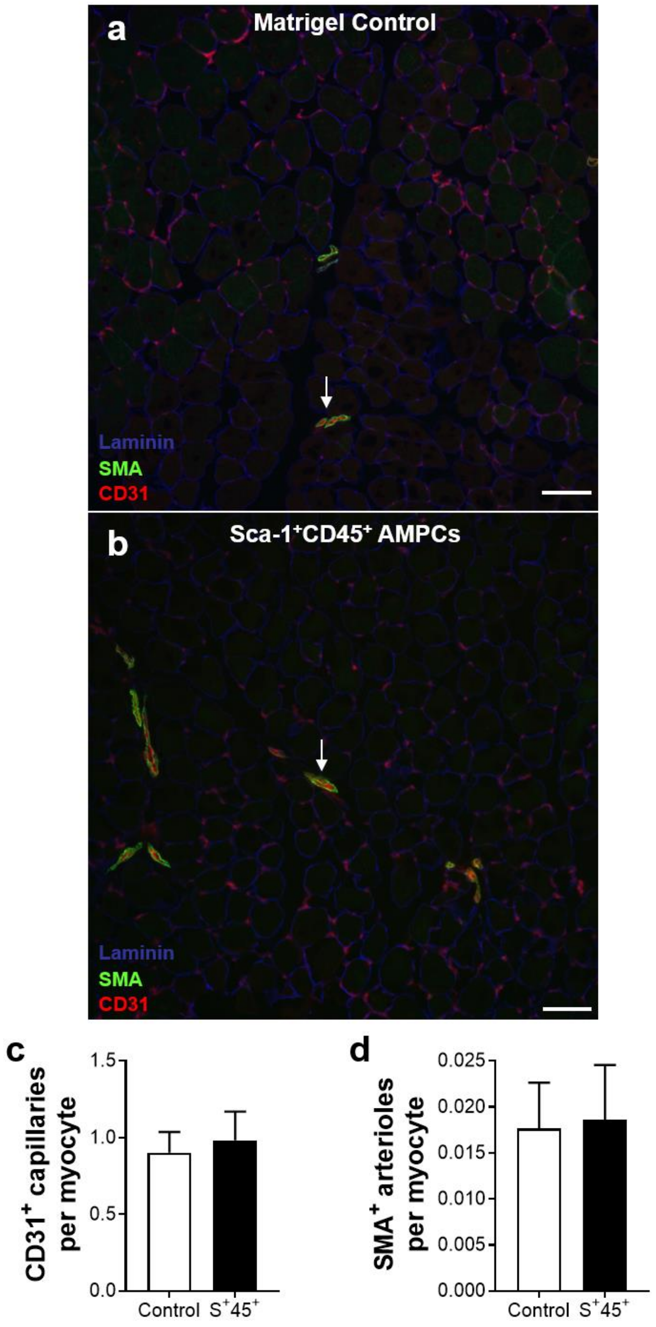

Supplementary Figure 9. Capillary and arteriolar density after cell transfer in hindlimb ischaemia model.

a, **b**, Examples of immunofluorescent images taken from sections of gastrocnemius muscle that had received injections of (a) cell-free Matrigel control or (b) Sca-1$^+$CD45$^+$ cells at the time of hindlimb ischaemia surgery. Examples of CD31$^+$SMA$^+$ arteriolar staining are indicated by arrows. Counter-staining was performed with an antibody to laminin to help

delineate myocytes and DAPI for nuclei. Scale bar: 100µm (white). **c**, **d**, Quantification of the (c) capillary and (d) arteriolar density from gastrocnemius sections for the two groups. Data are shown as mean±sd from n=5 mice per group. *P*-values were not significant by Mann-Whitney tests.

	$Sca\text{-}1^+CD45^+$	$Sca\text{-}1^+CD45^-$	P-value
$CD31^+$	81.3% (39.7 – 99.7)	17.8% (12.1 – 23.9)	0.06
$CD144^+$	87.8% (78.5 – 88.8)	37.7% (33.7 – 41.4)	0.25
$CD146^+$	60.6% (23.5 – 74.0)	4.3% (2.9 – 6.5)	0.06
$CD140a^+$	8.6% (5.8 – 17.9)	2.9% (1.1 – 5.8)	0.06
$LYVE1^+$	36.0% (25.6 – 41.6)	9.0% (8.5 – 12.2)	0.25
$F4/80^+$	33.9% (11.5 – 44.2)	1.9% (0.4 – 3.6)	0.06
$c\text{-}Kit^+$	28.8% (7.2 – 29.2)	2.2% (1.6 – 4.0)	0.25

Supplementary Table 1. Surface marker expression on cells gated from the Sca-1$^+$CD45$^+$ and Sca-1$^+$CD45$^-$ populations in aortic ring assays from C57BL/6 mice.

Shown are the median and range values for percent expression of different surface markers expressed by cells gated from the Sca-1$^+$CD45$^+$ or Sca-1$^+$CD45$^-$ subpopulations after vascular sprouting in the *ex vivo* aortic ring assay. n=3-5 C57BL/6 aortas used. Statistical comparisons were performed by Wilcoxon matched-pairs signed rank test.

**Antibody**	**Manufacturer**
AF488 mouse anti-TIE2	BD Phosflow
BUV395 anti-mouse CD45.2	BD Horizon
BV421 anti-mouse CD31	BD Horizon
BV605 anti-mouse FLK-1 (VEGFR2)	BD OptiBuild
BV786 anti-mouse Ly-6A/E (Sca-1)	BD Horizon
eFluor 660 anti-mouse Lyve-1	eBioscience
PE anti-mouse CD106	BD Pharmingen
PE-Cy7 anti-mouse CD144 (VE-cadherin)	BioLegend
PE-CF594 anti-mouse CD117 (c-Kit)	BD Horizon
AF488 anti-mouse CD140b (PDGFRb)	BD Phosflow
PE-Cy7 anti-mouse F4/80	BioLegend
BV605 anti-mouse CD146	BD OptiBuild
Other	
Fixable Viability Stain 700	BD Horizon
Mouse BD Fc Block	BD Pharmingen

Supplementary Table 2. Antibodies and reagents used for flow cytometry.

Antibody	Manufacturer
Controls	
D-galactose 0.4 M (control for Griffonia lectin)	
Goat IgG, polyclonal	Sigma, St Louis, MO
Mouse IgG, monoclonal	R&D Systems, Minneapolis, MN
Rabbit IgG, polyclonal	R&D Systems
Rat $IgG_{2a\kappa}$, monoclonal	BD Pharmingen, San Jose, CA
Rat $IgG_{2b\kappa}$, monoclonal	BD Pharmingen
Primary Antibodies	
Goat anti-mouse Sca-1	R&D Systems
Griffonia (Bandeiraea) Simplicifolia Lectin I Isolectin B4 Biotinylated	Vector Labs, Burlingame, CA
Mouse anti-human SMA (smooth muscle actin)	Dako, Carpinteria, CA
Rat anti-GFP	MBL, Woods Hole, MA
Rat anti-mouse CD31	BD Pharmingen
Rat anti-mouse CD45	BD Pharmingen
Rat anti-mouse MOMA-2	Abcam, Cambridge, MA
Rat anti-mouse Sca-1	BD Pharmingen
Rat anti-mouse Laminin α1/β1	Merck Millipore, Burlington, MA
Rat anti-Ly76 (Ter119)	Abcam
Rat anti-mouse CD31	Dianova, Hamburg, Germany
Rabbit anti-GFP	Life Technologies, Molecular Probes, Grand Island, NY
Rabbit anti-human vWF	Dako
Rabbit anti-Ki67	Abcam
Rabbit anti-mouse Lyve-1	Abcam
Rabbit anti-CD31 (polyclonal)	Abcam
Mouse anti-Actin, α-Smooth Muscle	Merck
Secondary Antibodies	
Donkey anti-goat AF488, AF647	Life Technologies
Donkey anti-rabbit AF647	Life Technologies
Donkey anti-rat AF488, AF594	Life Technologies
Donkey anti-mouse AF488	Life Technologies
Goat anti-mouse AF594, AF647	Life Technologies
Goat anti-rat AF488, AF594, AF647	Life Technologies
Goat anti-rabbit AF488, AF594, AF647	Life Technologies
Other	
Donkey serum	Sigma
Goat serum	Sigma
Hoechst	Sigma
DAPI	Life Technologies

Supplementary Table 3. Antibodies and reagents used for tissue immunofluorescent staining.

AF= Alexa Fluor.

Gene symbol	Gene name	Assay-on-demand reference
18s	18s	Mm03928990_g1
Ace	Angiotensin I converting enzyme (peptidyl-dipeptidase A) 1	Mm00802048_m1
Ccl11	Chemokine (C-C motif) ligand 11	Mm00441238_m1
Cd248	CD248 molecule, endosialin	Mm00547485_s1
Csf1	Colony stimulating factor 1 (macrophage)	Mm00432686_m1
Gapdh	Glyceraldehyde-3-phosphate dehydrogenase	Mm99999915_g1
Vegfa	Vascular endothelial growth factor A	Mm01281449_m1
Vegfc	Vascular endothelial growth factor C	Mm00437310_m1
Wnt5a	Wingless related MMTV integration site 5a	Mm00437347_m1

Supplementary Table 4. Genes used for RT-qPCR validation of microarray data.

All acquired from Life Technologies.
